# Rational Surface Engineering Reduces Advanced Glycation End‐Product Adsorption and Improves Implant Integration

**DOI:** 10.1002/advs.78507

**Published:** 2026-10-27

**Authors:** Thien Ngoc Le, Wenhao Chen, Tengxiao Huang, Tien Thanh Nguyen, Markos Negash Alemie, Xuan Duy Do, Giles Best, Jordan Li, Jitraporn Vongsvivut, Vi‐Khanh Truong, Weiwei Li, Yunpeng Zhao, Richard Bright, Krasimir Vasilev

**Affiliations:** ^1^ Biomedical Nanoengineering Laboratory College of Medicine and Public Health Flinders University South Australia Australia; ^2^ Department of Orthopaedics Qilu Hospital Cheeloo College of Medicine Shandong University Jinan Shandong People's Republic of China; ^3^ Flow Cytometry Facility Health and Medical Research Building College of Medicine and Public Health Flinders University South Australia Australia; ^4^ Department of Renal Medicine Flinders Medical Centre South Australia Australia; ^5^ Infrared Microspectroscopy (IRM) Beamline Australian Synchrotron Victoria Australia; ^6^ Department of Pathology Qilu Hospital Cheeloo College of Medicine Shandong University Jinan China

**Keywords:** advanced glycation end products, diabetes, implant integration, inflammation, macrophage, osteointegration, plasma polymerization, surface modification

## Abstract

Advanced glycation end‐products (AGEs) accumulate in metabolic diseases such as diabetes and are closely associated with poor outcomes following implantation. However, how these molecules influence events at the biomaterial interface has remained largely unknown. Here, we identify AGEs as critical biointerfacial modifiers that adsorb onto implant surfaces, reprogram immune responses, promote fibrotic encapsulation, impair angiogenesis, and compromise osseointegration. This suggests a potential mechanism by which metabolic disease disrupts biomaterial–tissue integration. By engineering titanium surfaces enriched with amine or carboxyl functionalities, we create AGE‐resistant biointerfaces that effectively suppress pro‐inflammatory M1 macrophage polarization, promote an anti‐inflammatory M2 macrophage phenotype, reduce fibrotic encapsulation, enhance angiogenesis, and improve osseointegration across ex vivo, in vitro, and in vivo diabetic models. These findings establish disease‐informed surface chemistry as a design paradigm for next‐generation implants intended for metabolically compromised and other high‐risk patient populations.

## Graphical abstract

Targeted surface chemical modification prevents the accumulation of advanced glycation end products (AGEs) on implant surfaces, attenuates inflammation and fibrosis, effectively restores angiogenic activity, and improves osseointegration under diabetic conditions.

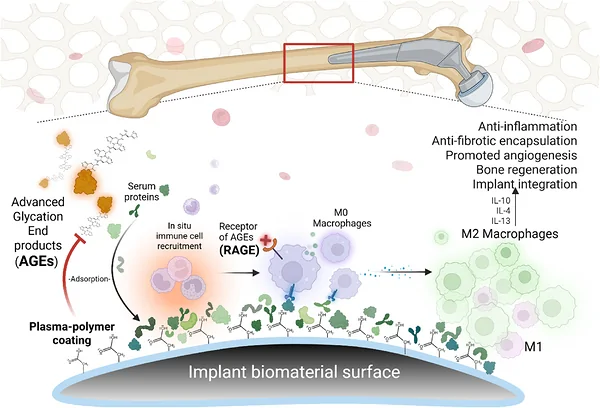

## 1 Introduction

Advanced glycation end products (AGEs) are covalent sugar adducts that form when excess glucose, fructose, or galactose in the bloodstream reacts with proteins and lipids [1]. The process that forms AGEs is called glycation, which, unlike glycosylation, does not involve any enzymatic event [2]. Instead, glycation occurs through complex, spontaneous chemical reactions, including rearrangement, dehydration, and condensation, forming multiple stable, heterogeneous AGEs [1]. AGEs are elevated in the blood and tissues of a significant proportion of the population, including people with diabetes, cardiovascular [3] and kidney disease [4], and those with a high body mass index (BMI). Circulating AGEs concentrations also increase with age [5]. For instance, in the diabetic population, glycated albumin (GA) has been measured at 30–40%, which is 2–3 times higher than in healthy individuals (11%–15%) [6]. In another study, the AGE blood concentration in type 2 diabetes (T2D) was found to be 6.6‐fold higher than in non‐diabetic subjects [7].

A high systemic AGEs burden is strongly linked to increased rates of post‐surgical complications [8]. For example, the complications with total hip arthroplasty were found to be up to 4 times higher in people with high BMI [9]. Significantly higher complication rates in the case of people with high BMI were documented with many other implants and procedures [10, 11]. In the case of diabetic patients, a 2023 study reported a 13.4 times higher complication rate with metatarsophalangeal joint arthrodesis [10]. In a large cohort study, Ovaska et al. reported a 120% increase in deep surgical site infections following ankle fracture surgery in diabetic patients [12]. Such complications can be devastating to patients, leading to amputations and patient death. In the case of dental implants, Wagner et al. reported a 43% higher risk for implant failure in patients with diabetes [13]. Regarding soft implants, Rifkin et al. reported that diabetic patients had 75% higher rates of surgical site infection in implant‐based breast reconstruction [14].

Numerous surface‐modification strategies, including nanoscale topographies [15], hydroxyapatite coatings [16], bioactive ions [17], and growth‐factor immobilization [18], have been developed to enhance implant biocompatibility and osteoconductivity. While these strategies can enhance osteoconductivity and biocompatibility under physiological conditions, their effectiveness may be compromised in patients with diabetes and other chronic inflammatory disorders because of elevated AGEs accumulation, persistent inflammation, and impaired tissue regeneration [19]. Plasma polymerization offers a versatile surface‐engineering strategy that enables precise control over interfacial chemistry, charge [20], wettability [21], stiffness [22], ligand presentation [23], and nanoscale architecture [24] without altering the bulk properties of the underlying implant. Plasma polymer coatings can regulate protein adsorption, modulate immune responses, promote tissue integration, and prevent infection [25, 26, 27, 28, 29]. Importantly, unlike many existing approaches, this strategy can be specifically designed to address the pathological molecular environment associated with AGE accumulation at the implant interface and mitigate downstream inflammatory responses that compromise implant integration. This capability represents a unique advantage for improving implant performance in patient populations at high risk of implant‐related complications.

Given that host responses are critically governed by the molecular identity established within the first moments after implantation [30], understanding whether AGEs participate in forming this biointerface is essential for developing rational, disease‐targeted biomaterial strategies [31].

Here, we hypothesize that AGEs act as pathological, surface‐active biointerface modifiers that adsorb onto implantable biomaterials and accumulate over time, establishing a disease‐specific interface that elicits immune dysfunction and macrophage reprogramming and ultimately drives inflammation, fibrosis, impaired angiogenesis, and compromised osseointegration. Importantly, we distinguish between soluble circulating AGEs and surface‐immobilized AGEs at the biomaterial interface, hypothesizing that the latter are sufficient to locally reprogram immune and regenerative responses independent of systemic AGE exposure.

We further propose that rational engineering of biomaterial surface chemistry can selectively modulate AGE adsorption, thereby reprogramming cell–material interactions in AGE‐rich environments. To test this second hypothesis, we selected acrylic acid (AC), allylamine (AA), and 1,7‐octadiene (OD) as plasma polymerization precursors because they generate chemically distinct surface functionalities and allow us to evaluate how a wide range of surface chemistries modulates AGE adsorption [32, 33]. AC produces carboxyl group (‐COOH)‐rich hydrophilic surfaces with a net negative surface charge [34]. AA generates amine group (‐NH_2_)‐rich surfaces that are positively charged under physiological conditions [35]. Both AA‐ and AC‐derived coatings are well documented as highly biocompatible and cytocompatible [25, 26, 36, 37]. In contrast, OD forms hydrocarbon‐rich, relatively hydrophobic surfaces [25]. The selected plasma polymer chemistries have been extensively characterized and utilized in biomaterials research and have been shown to differentially regulate protein adsorption, immune responses, tissue integration, and cellular behavior [28, 38, 39]. Their selection therefore provides a rational platform for determining how surface chemistry, wettability, and charge influence AGE accumulation and its biological consequences.

Using medical‐grade titanium as a clinically relevant model system, we combined human serum studies, in vitro immune cell assays, and in vivo diabetic implantation models to show that targeted amine‐ and carboxyl‐functionalized surfaces resist AGE accumulation while preserving favorable protein interactions. Our findings identify AGE adsorption as an unrecognized contributor to disease‐specific biomaterial failure and highlight surface chemistry design as a powerful strategy to modulate cell–material interactions in AGE‐rich environments.

## 2 Results

### 2.1 Surface Characterization

To test the hypotheses underpinning this study, we first tailored the surface chemistry of medical‐grade titanium (Ti) by plasma polymerization (Figure 1a) using three precursors: acrylic acid (AC), allylamine (AA), or 1,7‐octadiene (OD). Plasma polymerization was selected because it enables the rapid formation of conformal, nanoscale coatings on virtually any substrate material and geometry, including complex implant architectures [28, 32, 33]. The process is solvent‐free, completed within minutes, and requires a single deposition step, making it highly attractive for translational manufacturing. Importantly, plasma polymers are widely recognized as versatile and robust platforms for introducing stable chemical functionalities onto biomaterial surfaces, with extensive validation across numerous biomedical applications, including control of protein adsorption [23, 25, 28].

**FIGURE 1:**
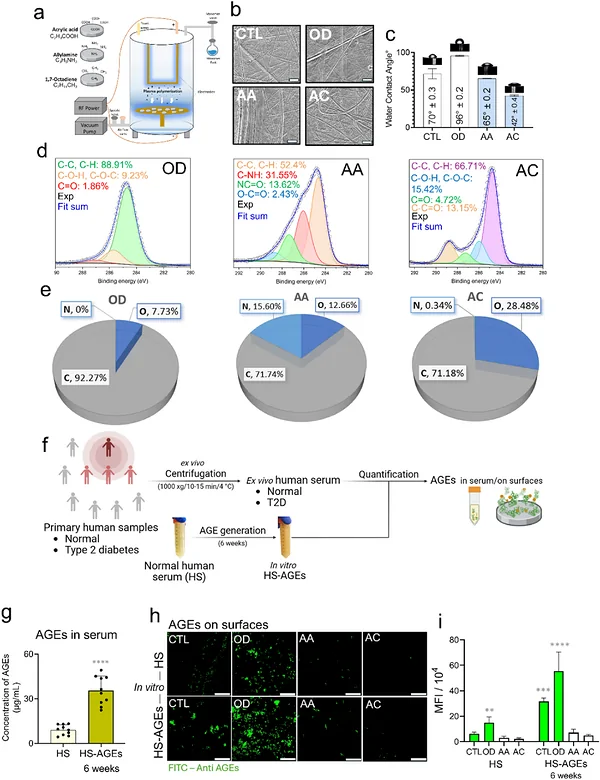
Surface engineering reduces the adsorption of AGEs. (a) Schematic diagram illustrating the non‐thermal plasma polymerization technique used to generate surfaces coated with acrylic acid (AC), allylamine (AA), and 1,7‐octadiene (OD). (b) Representative scanning electron microscopy (SEM) images of plasma polymer‐coated surfaces. (c) Sessile‐drop water contact‐angle measurements showing surface wettability. (d) XPS survey spectra of the C1s region for the three coatings. (e) Elemental composition of the surfaces by XPS in atomic percent. (f) Schematic representing ex vivo and in vitro experiments involved in quantifying advanced glycation end products (AGEs) in clinical serum and on surfaces. (g) FITC‐conjugated anti‐AGE antibody‐based quantification of AGEs in human serum (HS) and in vitro‐generated 6‐week AGEs (HS‐AGEs) serum (*n* = 10). (h) Confocal fluorescence microscopy images showing reduced AGEs accumulation on AA‐ and AC‐coated surfaces after incubation with in vitro‐generated AGEs and control serum from normal individuals. Scale bar represents 100 µm. (i) FITC‐conjugated anti‐AGE antibody‐based quantification of AGEs adsorbed on different surfaces after incubation with human pooled serum (HS) or in vitro‐generated 6‐week AGEs (HS‐AGEs) serum (*n* = 3). Data plotted are the mean ± standard deviation, *p < 0.05, **p < 0.01, ***p < 0.001 and ****p < 0.0001.

SEM imaging revealed scratch‐like features originating from the mechanical processing of the underlying titanium substrates. As all samples were prepared from the same titanium material using an identical substrate preparation procedure before plasma polymer deposition, these features were qualitatively comparable across all experimental groups (Figure 1b). The plasma polymer coatings did not appreciably change the underlying microscale surface morphology. Thus, while such substrate topography may contribute to the overall biomaterial response, it is common to all experimental groups and therefore does not account for the differences observed between the respective surface chemistries. The three precursors yielded coatings with water contact angles ranging from 42 to 95 degrees, with AC being the most hydrophilic and OD the most hydrophobic (Figure 1c). Chemical analysis by X‐ray photoelectron spectroscopy (XPS) revealed the presence of carbon (C 1s) and oxygen (O 1s) as the prominent components in all surface coatings, while nitrogen (N 1s) was only detected in the AA‐coated surface, consistent with the chemical composition of the precursors used (Figure 1e). The deconvoluted C1s high‐resolution spectra of the AC‐, AA‐, and OD‐coated surfaces revealed components corresponding to various carbon chemical states, with oxygen‐bonded carbon most abundant on the AC‐modified Ti surface, consistent with the highest hydrophilicity of these coatings (Figure 1d). Together, these surface modifications provided four distinct, well‐characterized model platforms for assessing AGE surface adsorption and accumulation and the subsequent biological responses. The stability of the plasma polymer coatings is an important consideration for their potential biomedical application. The coatings used in this study are typically highly crosslinked and adhere strongly to underlying substrates via chemical and physical bonds formed during energetic plasma deposition. Our recently published study showed no significant change in coating thickness after 24 h of incubation in phosphate‐buffered saline (PBS) at 37°C, indicating no detectable delamination or dissolution [40].

To assess the longer‐term stability of the plasma polymer coatings, samples were incubated in PBS at 37°C for 28 d and analyzed longitudinally by ellipsometry, water contact angle measurements and XPS. Coating thickness decreased only modestly over this period, from 32.77 to 30.35 nm for OD, 27.49 to 25.79 nm for AA, and 12.64 to 10.75 nm for AC (Figure S1), without evidence of abrupt film loss. Wettability showed similarly modest changes, while the characteristic surface ordering (OD > CTL > AA > AC) was maintained throughout the 28‐day period (Figure S2). XPS analysis confirmed that the characteristic surface chemical compositions persisted after prolonged incubation, with relatively small increases in oxygen content consistent with gradual surface oxidation (Figure S3). Collectively, these results demonstrate that the plasma polymer coatings remain structurally and chemically stable over 28 d under prolonged aqueous exposure, with only limited aging‐related changes and no evidence of coating delamination or extensive degradation.

### 2.2 Surface Chemistry Modulates AGE Accumulation at the Biomaterial Interface

Figure S4 illustrates the signaling pathways of AGE‐associated inflammatory responses. To evaluate whether surface chemistry is able to modulate the accumulation of AGEs, we first generated AGEs in vitro (HS‐AGEs) by incubating pooled human serum (HS) with high glucose for 6 weeks (Figure S5a). This process produced the characteristic yellow–brown coloration (Figure 1f). The concentration of AGEs in HS‐AGEs increased fourfold to 35.58 ± 9.12 µg mL^−1^, compared with HS (9.09 ± 3.22 µg mL^−1^) (Figure 1g). Next, we incubated native Ti controls and plasma‐polymer‐coated Ti samples with different chemical modifications in HS and HS‐AGEs. The adsorption of AGEs was visualized using a FITC‐conjugated anti‐AGE antibody‐based assay, as depicted in Figure 1h. The results showed a dramatic reduction in surface‐adsorbed AGEs on the AA and AC coatings compared with the CTL and OD coatings (Figure 1i). We repeated these experiments with clinical T2D samples. The clinical T2D cohorts had more than 10 years of disease duration (Table S3) and, as expected, AGE levels were significantly elevated compared with normoglycemic participants (*p* < 0.0001; Figure S5b and Table S1). Furthermore, validation of these findings was carried out using glycated albumin (HSA‐AGEs and BSA‐AGEs), which yielded similar outcomes (Figure S5c,d). To determine whether the reduced accumulation of AGEs on AA and AC surfaces resulted from a general reduction in protein adsorption, total serum‐protein adsorption was quantified using a Micro BCA assay. Following incubation with normal human serum, no significant differences in total adsorbed protein were detected between CTL, OD, AA and AC surfaces (*p* > 0.05; Figure S5e). Thus, the AA and AC coatings did not globally suppress serum‐protein adsorption. In contrast, AGE‐specific analyses demonstrated markedly lower accumulation of AGE‐modified proteins on AA and AC surfaces compared with CTL and OD across diabetic patient serum and independent glycated albumin models (*p* < 0.01; Figure S5f,g). These findings indicate that the reduced AGE accumulation on AA and AC surfaces is not simply a consequence of reduced total protein adsorption but reflects differential interactions between AGE‐modified proteins and the engineered surface chemistries.

### 2.3 Synchrotron Macro‐ATR‐FTIR Mapping of AGEs Adsorption

Synchrotron macro‐ATR‐FTIR microspectroscopy (Figure 2) was used to analyze AGEs adsorption patterns, revealing characteristic glycated protein peaks at 1053, 1088, 1374, 1492, 1526 cm^−^^1^. Principal component analysis (PCA) revealed distinct clustering for AA and AC surfaces, consistent with reduced adsorption of glycated components. To assess changes in biochemical interaction, we analyzed the key spectral regions (Figure 2a), including 1800–1700 cm^−1^ (ν(C═O) stretching vibration of lipids), 1685–1630 cm^−1^ (amide I band attributed to ν(C═O) stretching vibration of proteins), 1640–1600 cm^−1^ (amide II band associated with δ(N─H) bending and ν(C─N) stretching vibrations in R─CONH_2_ of amino acid structure), 1220–1350 cm^−1^ (amide III representing a combination of ν(C─N) stretching vibrations, δ(C─O) and δ(N─H) deformations). The saccharide exhibited five characteristic peaks (i.e., 1152, 1106, 1035, 1080 and 992 cm^−1^). Three distinct saccharide‐specific peaks (1094, 1106, and 1035 cm^−1^) were detected in HS‐AGEs adsorbed on both the modified and control surfaces. Our results indicate that the strong spectral overlap observed in the 1374–1053 cm^−1^ range is characteristic of GA. The heat maps depict the spatial variation in proteins (amide I, II, and III), glucose, and GA components across the surfaces (Figure 2b), with red and blue indicating the highest and lowest intensities of these components in HS‐AGEs, respectively. PCA revealed clear separation between AA/AC surfaces and CTL/OD surfaces, consistent with reduced adsorption of glycated protein components on AA and AC coatings (Figure 2c–f). Principal component analysis (PCA) scores for PC‐1 and PC‐2 showed that PC‐1 captured the dominant variance (78%, 82%, 95%, and 63%), indicating a strong influence of AGEs. The dispersed clustering on modified surfaces suggests transformation of glycated components. Table S2 summarizes these results.

**FIGURE 2:**
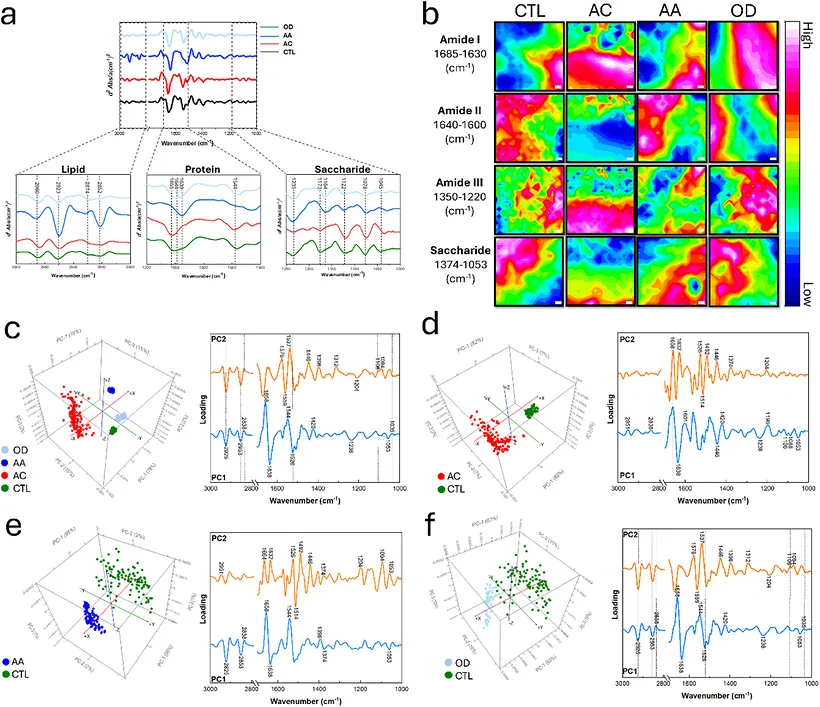
Synchrotron macro ATR‐FTIR biochemical changes upon adsorption of AGEs on biomaterial surfaces. (a) Comparative PCA score plots showing lipid, protein, and carbohydrate variation in the corona across four surfaces. (b) The heat maps reflect the variation of the protein Amide I (1685–1630 cm^−1^), Amide II (1640–1600 cm^−1^), Amide III (1350–1220 cm^−1^) and saccharide (1374–1053 cm^−1^) levels of the specific substrates on the surfaces (CTL, AC, AA and OD). Scale bars correspond to 5 µm. Red indicates high intensity, and blue shows low intensity. Table S2 lists the peaks of interest and their corresponding assignments. (c) PCA of the control and three modified surfaces (left). Loading spectra correlations of PC‐1 and PC‐2 scores for the control and three modified surfaces (right). (d) PCA of the AC and CTL surfaces (left). Loading spectra correlations of PC‐1 and PC‐2 scores for the AC and CTL surfaces (right). (e) PCA of the AA and CTL surfaces (left). Loading spectra correlations of PC‐1 and PC‐2 scores for the AA and CTL surfaces (right). (f) PCA of the OD and CTL surfaces (left). Loading spectra correlations of PC‐1 and PC‐2 scores for the OD and CTL surfaces (right).

### 2.4 Surface Chemistry‐Dependent Cytocompatibility, Macrophage Polarization, and ROS Generation

To determine whether the inflammatory response associated with AGE‐bearing surfaces could instead be explained by soluble AGE‐modified species released into the medium, macrophages were exposed to conditioned medium collected after 24 h incubation of AGE‐pre‐adsorbed Ti surfaces. No significant differences in *TNF‐α, IL‐1β* or *IL‐6* were observed between macrophages treated with conditioned medium from AGE‐pre‐adsorbed surfaces and those treated with control conditioned medium (Figure S9). In contrast, direct macrophage contact with AGE‐bearing surfaces produced the inflammatory responses described above. These findings demonstrate that soluble species released from the AGE‐pre‐adsorbed surfaces were insufficient to reproduce the inflammatory phenotype and strongly support a predominantly interface‐dependent mechanism.

The cytocompatibility of the biomaterial surfaces after incubation in normal serum, serum from T2D patients, and in vitro‐generated AGEs was evaluated using human THP‐1 monocytes and THP‐1‐derived macrophages (dTHP‐1). Annexin V/PI staining and flow cytometry analyses confirmed that the AA‐ and AC‐coated surfaces supported the highest cell viabilities under both ex vivo and in vitro AGE‐rich conditions (80–92%), whereas OD coatings consistently reduced viability (73%–87%) and increased apoptosis (9%–19%) (Figure S6a–c). The proportion of early apoptotic and dead THP‐1 cells was also significantly lower on the AA‐ and AC‐coated surfaces (*p* < 0.01). Similar trends were observed in dTHP‐1 macrophages (Figure S6d–f).

Next, we assessed macrophage polarization (Figure 3a). After incubation in normal or AGE‐rich serum from T2D patients (Figure 3) or in in vitro‐generated serum (Figure S7), the AA and AC coatings promoted anti‐inflammatory M2 differentiation, whereas CTL and OD surfaces favored pro‐inflammatory M1 responses. Morphological changes were evident: M2 macrophages were larger and more irregular, with multiple filopodia (Figure S7a). Flow cytometry confirmed reduced CD80^+^ M1 macrophages on AA (35.3 ± 4.1%) and AC (20.2 ± 1.3%) compared with CTL (53.0 ± 1.1%) and OD (66.3 ± 4.4%), *p* < 0.001, while CD206^+^ M2 macrophages were significantly increased on the AC (68.2 ± 2.4%) and AA (54.4 ± 0.7%) vs CTL (50.7 ± 1.5%) and OD (33.3 ± 0.7%), *p* < 0.0001 (Figure 3b,c; Figure S7b,c). Macrophages cultured on AA and AC surfaces and incubated in T2D patient serum also showed markedly reduced ROS levels (*p* < 0.001) (Figure 3d,e).

**FIGURE 3:**
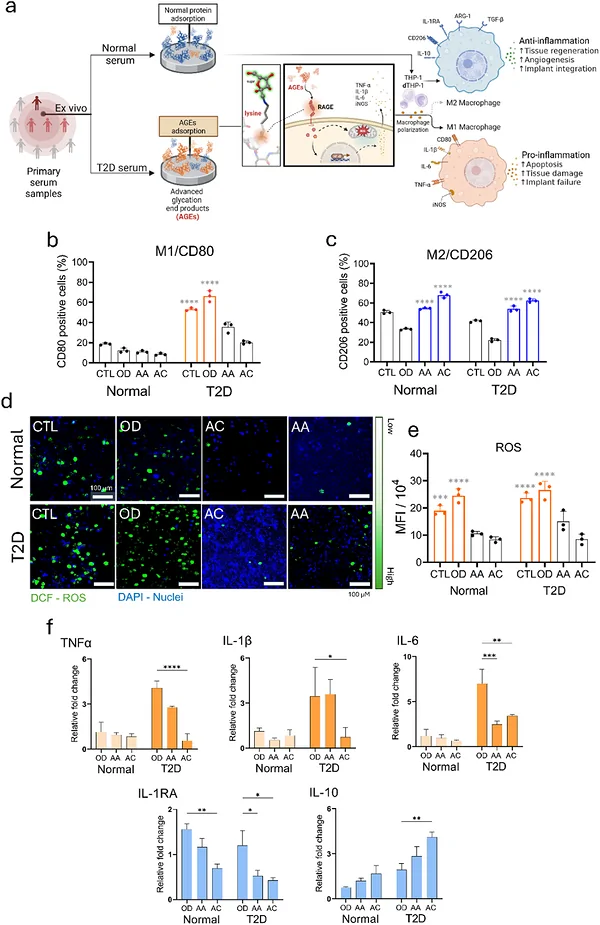
Reduced AGE accumulation on the engineered surfaces is associated with enhanced anti‐inflammatory responses. (a) Schematic representation of protein adsorption and immune responses of THP‐1 monocytes and dTHP‐1 macrophages post‐incubation in ex vivo serum from primary cohorts (normal and T2D). (b) Flow cytometry analysis of CD80^+^ M1 macrophages on the functional surfaces pre‐adsorbed with ex vivo serum from primary cohorts. (c) Flow cytometry analysis of CD206^+^ M2 macrophages on the functional surfaces pre‐adsorbed with ex vivo serum from primary cohorts. (d) Confocal microscopy images of macrophages cultured on surfaces pre‐adsorbed with ex vivo serum, showing intracellular ROS (DCF, green) and nuclear staining (DAPI, blue); scale bar: 100 µm. (e) ROS quantification by mean fluorescence intensity (MFI). (f) Gene expression showing fold changes in target genes, including pro‐inflammatory cytokines (*TNF‐α, IL‐1β, IL‐6*) and anti‐inflammatory cytokines (*IL‐10, IL‐1RA*), produced by macrophages cultured on surfaces pre‐adsorbed with serum from patient samples. Gene expression is presented as fold change relative to the CTL titanium group, which served as the normalization reference (fold change = 1). Data are presented as mean ± SD, *n* = 3, *p < 0.05, **p < 0.01, ***p < 0.001 and ****p < 0.0001.

On AA‐ and AC‐coated surfaces, reduced AGEs adsorption was associated with lower gene expression of *TNF‐α, IL‐1β*, *IL‐6*, and *iNOS* (Figure 3f and Figure S8g), alongside elevated expression of the anti‐inflammatory markers *ARG‐1*, *TGF‐β*, and *IL‐10* (Figure 3f and Figure S8h). In contrast, OD surfaces elicited the opposite response, with increased AGE accumulation driving sustained pro‐inflammatory cytokine production. The results were consistent across surfaces incubated with either T2D patient serum or HS‐AGEs serum. Expression of CD80^+^ (an M1 marker) and CD206^+^ (an M2 marker) in macrophages was used to gate the M1‐like (Figure S8c) and M2‐like (Figure S8e) cells between the study groups (HS and HS‐AGEs groups). The proportion of M1‐like macrophages was significantly greater in HS‐AGEs than in normal HS (Figure S8d). AA‐ and AC‐coated surfaces significantly reduced M1‐like cells in both the normal HS group (*p* < 0.01) and the HS‐AGEs group (*p* < 0.0001). Conversely, the two hydrophilic‐modified surfaces stimulated macrophage activation toward an anti‐inflammatory (M2‐like) phenotype (Figure S8f). CTL and hydrophobic OD‐coated surfaces showed a significantly lower percentage of these M2‐like cells in both HS and HS‐AGEs (*p* < 0.0001). AA and AC coatings enhanced M2‐like macrophage activation even under AGE‐rich conditions (AC 55.8%, AA 45.37% vs OD 26.56% and CTL 34.14% of M2‐like cells; *p* < 0.0001).

Next, we examined how surface‐adsorbed AGEs affect oxidative stress generation (Figure S8a). Notably, intracellular reactive oxygen species (ROS) levels in macrophages were markedly reduced on AA‐ and AC‐coated surfaces pre‐adsorbed with HS and HS‐AGEs compared with CTL‐ and OD‐coated surfaces. Quantification of the fluorescence intensity is presented in Figure S8b. Hydrophobic OD coating exhibited a significant increase in ROS levels of macrophages, which were exposed to both pre‐adsorbed proteins from HS and HS‐AGEs (HS, OD vs AC, *p* < 0.0001; OD vs AA, *p* < 0.0001; OD vs CTL, *p* < 0.001; HS‐AGEs, OD vs AC and AA, *p* < 0.0001; OD vs CTL, *p* < 0.001). In the AGE‐rich condition, the modified amine‐ and carboxyl‐rich coatings significantly reduced ROS expression in activated macrophages (*p <* 0.001). AA and AC coatings reduce AGEs adsorption, preserve macrophage viability, and promote anti‐inflammatory M2 polarization, supporting their potential for AGE‐resistant implants in diabetic settings. AA and AC surfaces reduced AGE adsorption without compromising overall protein compatibility, as shown by preserved cell viability, reduced oxidative stress, and enhanced pro‐healing macrophage responses.

We observed no significant differences in *TNF‐α, IL‐1β*, or *IL‐6* gene expression (Figure 3f) for ex vivo samples, and in *TNF‐α, IL‐1β, IL‐6, or iNOS* responses (Figure S8g) for in vitro samples, suggesting limited release of biologically active AGEs under the tested conditions. AA and AC coatings showed significantly increased anti‐inflammatory responses in *IL‐10* and *ARG‐1* (*p* < 0.01) for both ex vivo (Figure 3f) and in vitro samples (Figure S8h) exposed to AGE‐rich conditions.

These findings show that limiting AGE adsorption through rational surface chemistry preserves macrophage viability, reduces oxidative stress, and promotes pro‐regenerative immune polarization, making AA and AC coatings promising candidates for AGE‐resistant implant interfaces in diabetic settings.

### 2.5 AA and AC Coatings Reduce AGE‐Driven Fibrosis In Vivo

Excessive production of fibrotic growth factors and collagen deposition, driven by AGEs, promotes tissue fibrosis and impairs healing in diabetic patients [41]. In such a scenario, fibroblast adhesion activates fibroinflammatory mediators, leading to poor tissue regeneration after implantation.

Longitudinal monitoring confirmed maintenance of the diabetic phenotype throughout the 30‐day implantation period. Fasting blood glucose remained markedly elevated in STZ‐induced diabetic rats relative to healthy controls at all measured time points, without evidence of spontaneous normalization during follow‐up (Table S4; Figure S10). Circulating AGE levels were also consistently higher in diabetic animals than in healthy controls, including at the 30‐day endpoint (Table S5; Figure S10). These data confirm that the implants in diabetic animals remained exposed to sustained hyperglycemia and an elevated systemic AGE burden throughout the experimental period.

Building on our in vitro data, we systematically evaluated how biomaterial surface modifications affected fibrosis and angiogenesis after implantation in healthy and diabetic rats. We also used surfaces pre‐incubated with in vitro‐generated AGEs (AGEs (+)) as additional controls, following the protocols described above.

Ti capsules, coated with OD, AA, AC, and CTL, were surgically implanted in the dorsolateral region of rats (Figure 4a). In healthy rats, the fibrous layer on OD‐coated surfaces and CTL was significantly thicker than that on AA or AC coatings, regardless of whether they were pre‐incubated with serum containing AGEs (*p* < 0.0001) (Figure 4b). In diabetic rats, the anti‐fibrotic effect of the AA and AC coatings was substantially more pronounced, as these coatings reduced fibrous capsule thickness nearly two‐fold compared to the uncoated Ti control and three‐fold compared to OD (CTL: 133.56 µm, OD: 276.9 µm, AA: 88.1 µm, AC: 78.1 µm). Histological analysis with hematoxylin and eosin (H&E) and Masson's trichrome staining corroborated these findings, revealing markedly lower immune cell infiltration and collagen deposition around AA‐ and AC‐coated Ti capsules than around CTL and OD (Figure 4c,d). Together, these results indicate that the AA and AC coatings protect against AGE‐driven fibrosis.

**FIGURE 4:**
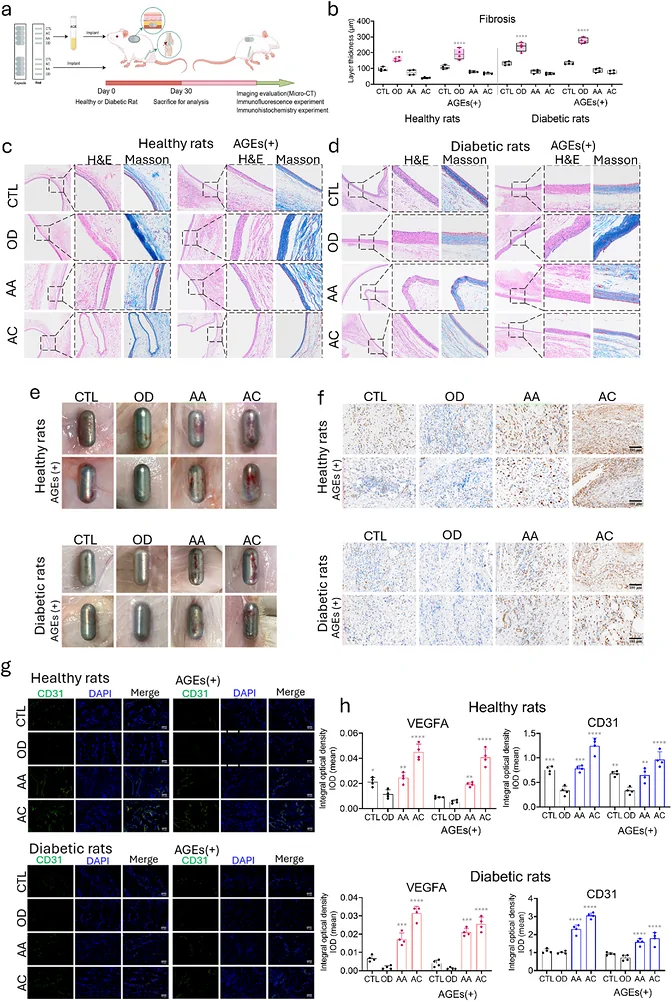
AGE resistance prevents fibrosis and promotes angiogenesis post‐implantation. (a) Schematic design detailing the process of surgical implantation of capsules and rods with different coatings in healthy and streptozotocin (STZ)‐ induced diabetic rats, with or without pre‐adsorption of AGEs. (b) Quantification of the fibrous capsule thickness four weeks post‐implantation (*n* = 4). (c) Histological evaluation of inflammatory cell infiltration (H&E) and collagen deposition (Masson's trichrome) in peri‐implant tissue from healthy rats (*n* = 6). Scale bars: 100 µm (main), 30 µm (inset). (d) Histological evaluation of inflammatory cell infiltration (hematoxylin and eosin, H&E) and collagen deposition (Masson's trichrome) in peri‐implant tissue from diabetic rats (*n* = 6). Scale bars: 100 µm (main), 30 µm (inset). (e) Macroscopic images of the implant surfaces in normoglycemic and diabetic rats (scale bar represents 5 mm) (*n* = 6). (f) Immunohistochemical staining for *VEGFA* in peri‐implant tissue (scale bar: 100 µm) (*n* = 6). (g) Immunofluorescence staining for CD31 in peri‐implant tissue (green: CD31; blue: nuclear DAPI; scale bar represents 50 µm, *n* = 6). (h) Quantitative analysis of *VEGFA* and CD31 expression in peri‐implant tissue from healthy and diabetic rats (*n* = 4). Data are presented as mean ± SD, *p < 0.05, **p < 0.01, ***p < 0.001 and ****p < 0.0001.

### 2.6 AGE‐Modulating Surfaces Restore Angiogenesis under Diabetic Conditions

The blood supply is vital for implant integration, but AGEs can impair it through endothelial dysfunction, which thickens vessel walls, triggers inflammation, and inhibits angiogenesis [42]. For this reason, we next examined angiogenesis. Macroscopic inspection revealed robust vessel formation and enhanced blood circulation around the AA‐ and AC‐coated implants (Figure 4e). Immunohistochemical staining for vascular endothelial growth factor‐α (VEGFA), a marker of vascular endothelial cell function and pro‐angiogenesis, showed significantly higher expression adjacent to AA and AC coatings than in CTL and OD (Figure 4f). Immunofluorescence for the endothelial marker cluster of differentiation 31 (CD31) further confirmed increased neovascularization around the AA‐ and AC‐coated implants in both healthy and diabetic rats (Figure 4g,h). Importantly, diabetic animals with uncoated Ti or OD‐coated implants showed markedly suppressed *VEGFA* and CD31 expression, consistent with the inhibitory effects of AGEs on angiogenesis. In contrast, AA and AC coatings produced a multi‐fold increase in *VEGFA* and CD31 expression, effectively restoring angiogenic activity under diabetic conditions to levels comparable to healthy animals implanted with uncoated Ti. Collectively, these results show that the AA and AC coatings attenuate fibrosis while promoting angiogenesis, two critical features required for successful implant integration. This dual functionality highlights their strong translational potential for implantable devices, particularly for patients with diabetes.

### 2.7 AGE‐Modulating Surfaces Enhance Osseointegration in Diabetic Bone

To assess in vivo osseointegration, Ti implants coated with OD, AA, or AC were surgically placed in the femoral condyles of diabetic rats. At 30 d post‐implantation, we harvested femurs for multimodal assessments, including micro‐computed tomography (micro‐CT) to examine bone microarchitecture and histology, immunofluorescence, and immunohistochemistry to assess osteogenesis. We used a computer‐controlled universal testing machine to conduct biomechanical testing and evaluate bone‐implant contact (BIC) strength (Figure 5a). In healthy rats, the AA‐ and AC‐coated surfaces showed the highest pull‐out force, regardless of AGEs pre‐adsorption (Figure 5b). In diabetic rats, the pull‐out force decreased overall. However, the AC coating yielded the highest pull‐out force, exceeding that of a healthy animal implanted with an uncoated Ti device.

**FIGURE 5:**
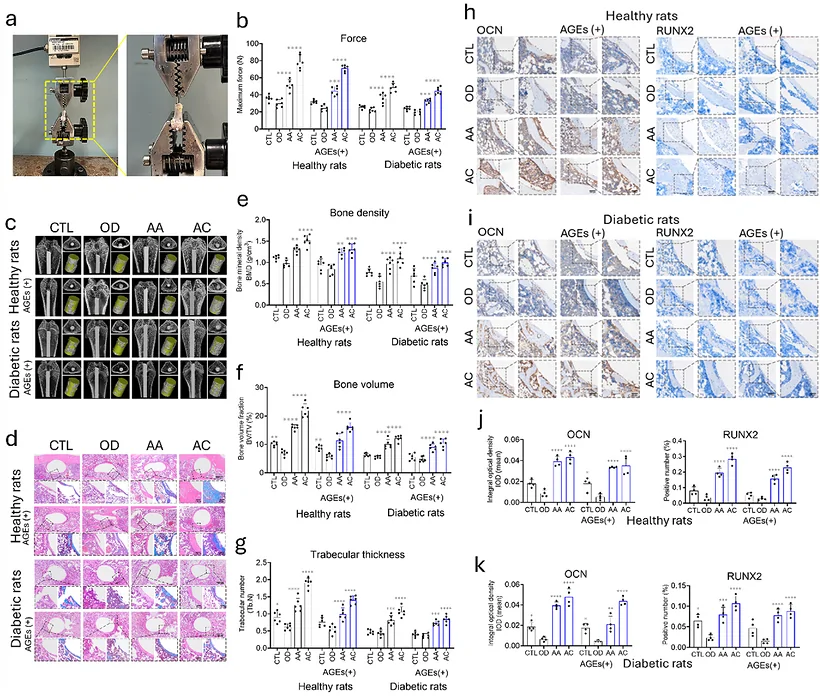
Improved osseointegration on AGE‐modulating surfaces. (a) Pull‐out biomechanical testing to evaluate bone‐implant contact strength. (b) Quantification of pull‐out forces under the study conditions for healthy and diabetic rats (*n* = 6). (c) Micro‐CT reconstructions (coronal, transverse, and 3D views) of femoral condyles with implants in healthy and diabetic rats (*n* = 6). (d) H&E‐stained femoral bone sections at 30 d post‐implantation (*n* = 6). (e) Bone mineral density (BMD) measurement after 30 d post‐implantation (*n* = 6). (f) Bone volume fraction (BV/TV) measurement post‐implantation (*n* = 6). (g) Trabecular number (Tb.N) measurement post‐implantation (*n* = 6). (h) Immunohistochemical staining for *OCN* and *RUNX2* expression in femoral bone tissues from healthy rats (*n* = 4). (i) Immunohistochemical staining for *OCN* and *RUNX2* expression in femoral bone tissues from diabetic rats (*n* = 4). (j) Immunohistochemical staining for *OCN* and *RUNX2* expression in femoral bone tissues from healthy rats (*n* = 4). (k) Immunohistochemical staining for *OCN* and *RUNX2* expression in femoral bone tissues from diabetic rats (*n* = 4). Data are presented as mean ± SD, *p < 0.05, **p < 0.01, ***p < 0.001 and ****p < 0.0001.

Micro‐CT analysis at day 30 revealed limited new bone formation and poor implant adhesion with CTL and OD coatings, whereas AA‐ and AC‐coated implants showed robust new bone development along the implant–tissue interface (Figure 5c). H&E staining (Figure 5d) and quantitative analysis within the 200 µm peri‐implant zone confirmed significantly improved bone mineral density (BMD; Figure 5e), trabecular number (Tb.N; Figure 5f), and bone volume fraction (BV/TV; Figure 5g) for the AA and AC coatings compared with CTL and OD. These osteogenic parameters were higher in healthy than diabetic rats, but the AA and AC coatings consistently outperformed CTL and OD in both conditions. Further immunohistochemical analyses confirmed significantly elevated expression of osteocalcin (OCN) and RUNX2 around the AA‐ and AC‐coated implants compared with CTL and OD (Figure 5h–k), underscoring the ability of these surfaces to stimulate osteogenesis even in diabetic conditions. These findings show that AA and AC coatings enhance bone–implant contact, biomechanical stability, and osseointegration under diabetic and AGE‐rich conditions.

### 2.8 AGE‐Modulating Surfaces Promote Pro‐Healing Immune Remodeling In Vivo

At the implant‐tissue interface, successful integration requires a transition of the innate immune response from a pro‐inflammatory to an anti‐inflammatory state, enabling functional tissue remodeling and implant tolerance. In diabetic patients, this process is often disrupted by greater immune cell recruitment, prolonged inflammation, and excessive fibrotic encapsulation [43]. The in vitro macrophage studies discussed above indicate the benefits of this remodeling process provided by the AC and AA coatings under AGE‐rich conditions. Consistent with in vitro findings, AA‐ and AC‐coated implants promoted M2 polarization and reduced M1 polarization in both healthy and diabetic animals, as confirmed by increased CD206^+^ and reduced *iNOS* expression in vivo (Figure 6).

**FIGURE 6:**
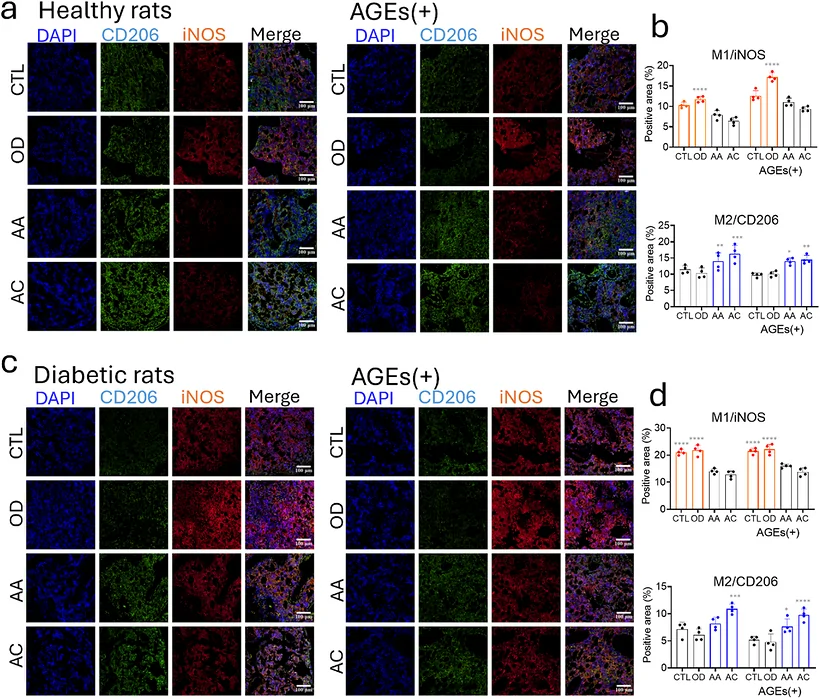
Advanced surface coatings aid anti‐inflammatory macrophage polarization. (a) Immunofluorescence staining for CD206^+^ (green) and *iNOS* (red) co‐staining in normoglycemic rats (*n* = 6). (b) Proportion of *iNOS*‐positive and CD206^+^‐positive area in normoglycemic rats (*n* = 6). (c) Immunofluorescence staining for CD206^+^ (green) and *iNOS* (red) co‐staining in diabetic rats (n = 6). (d) Proportion of *iNOS*‐positive and CD206^+^‐positive area in diabetic rats (*n* = 6). The scale bar represents 100 µm. Data are presented as mean ± SD. *p < 0.05, **p < 0.01, ***p < 0.001 and ****p < 0.0001.

### 2.9 AA and AC Coatings Attenuate Peri‐Implant Inflammation In Vivo

To further characterize the inflammatory response, we performed immunohistochemical staining of bone–implant interfaces at day 30. This demonstrated increased expression of the anti‐inflammatory cytokine *IL‐10* and decreased levels of the pro‐inflammatory cytokine *TNF‐α* in peri‐implant tissue from both healthy (Figure 7a,b) and diabetic rats (Figure 7c,d), and in the presence of AGEs (Figure S11). Histological evaluation of the subcutaneous tissues surrounding the implants also revealed significantly fewer infiltrating inflammatory cells and reduced inflammatory reactions with AA and AC coatings, consistent with their anti‐fibrotic effects.

**FIGURE 7:**
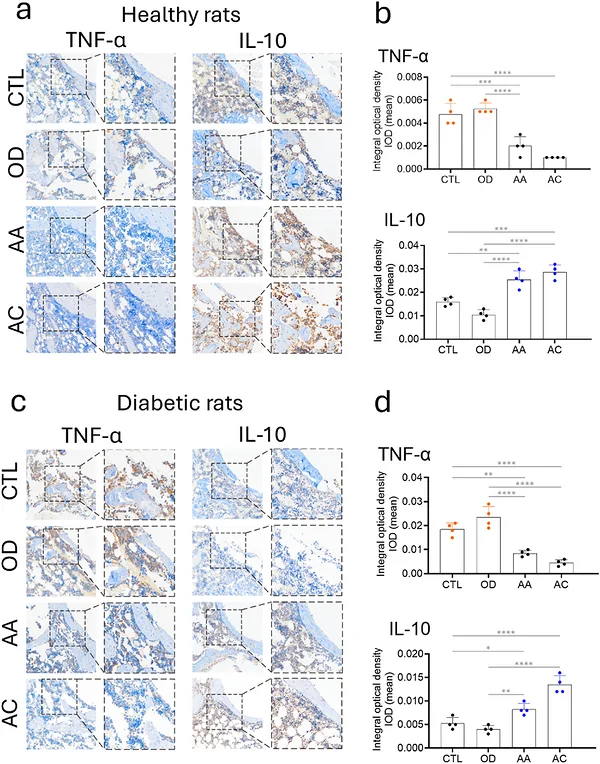
Advanced surface coatings aid anti‐inflammatory responses in peri‐implant bone tissue of healthy and diabetic rats. (a) Representative images of bone tissue from normoglycemic rats and (c) diabetic rats stained with antibodies to *TNF‐α* and *IL‐10*. Scale bars represent 50 µm. (b) Quantification of *TNF‐α* and *IL‐10* expression in healthy rats (*n* = 4). (c) Representative images of bone tissue from diabetic rats stained with antibodies to *TNF‐α* and *IL‐10*. Scale bars represent 50 µm. (d) Quantification of *TNF‐α* and *IL‐10* expression in diabetic rats (*n* = 4). Data are presented as mean ± SD. *p < 0.05, **p < 0.01, ***p < 0.001 and ****p < 0.0001.

## 3 Discussion

Metabolic diseases profoundly alter the biological milieu into which medical devices are implanted, yet biomaterial design has historically focused on universal solutions rather than patient‐specific pathology [44]. Among the molecular hallmarks of diabetes and related conditions, AGEs are strongly associated with impaired healing, chronic inflammation, fibrosis, and vascular dysfunction [9, 45]. Despite decades of clinical evidence linking high AGE burden to poor surgical outcomes [46], it remains unresolved whether these molecules directly shape the biomaterial interface.

Here, we demonstrate that AGEs are not merely circulating biomarkers of disease but rather act as surface‐active pathological modifiers that adsorb onto implant materials and govern subsequent immune and regenerative events. This finding provides a mechanistic bridge between systemic metabolic dysfunction and local implant failure. By forming part of the initial molecular surface‐adsorbed protein layers, AGEs reprogram macrophage behavior toward sustained inflammation [47], increase oxidative stress [48], promote fibrotic encapsulation [49], suppress angiogenesis [50], and ultimately impair osseointegration [51].

The distinction between soluble and surface‐associated AGEs was further examined using conditioned‐medium experiments. Medium collected after 24 h incubation of AGE‐pre‐adsorbed titanium surfaces did not significantly increase *TNF‐α, IL‐1β*, or *IL‐6* in macrophages, whereas direct interaction with AGE‐bearing surfaces elicited a clear inflammatory response. These findings argue against desorbed soluble AGEs being the principal driver of the observed phenotype and support the concept that AGE accumulation at the material interface creates a distinct local biological stimulus. This interfacial mechanism is therefore mechanistically different from the classical paradigm in which circulating soluble AGEs activate cells through ligand–RAGE interactions alone.

Our results show that controlling AGE adsorption on surfaces is a powerful way to interrupt this cascade. This work demonstrates that rational chemical functionalization of titanium can markedly limit AGE accumulation, even in highly glycated environments derived from patients with long‐standing diabetes. Amine‐ and carboxyl‐rich plasma polymer coatings consistently resisted AGE deposition across in vitro glycated serum, purified glycated albumin, and clinical samples. Importantly, reduced AGE accumulation was associated with preserved macrophage viability, suppressed ROS generation, and promoted polarization toward pro‐healing phenotypes.

Notably, the reduced AGE accumulation observed on AA and AC surfaces was not associated with a general reduction in serum‐protein adsorption. Micro‐BCA analysis demonstrated comparable total protein adsorption across all surface chemistries following exposure to normal serum, whereas AGE‐specific measurements consistently showed substantially lower accumulation of AGE‐modified proteins on AA and AC. Together with the preserved cell adhesion and viability observed on these surfaces, these findings argue against a nonspecific antifouling mechanism. Instead, they support the idea that surface chemistry alters the composition of the interfacial protein layer, limiting enrichment of AGE‐modified proteins while still adsorbing serum proteins compatible with cellular attachment and function.

Importantly, the biological relevance of the differential AGE–surface interactions observed using clinical serum was further supported by the in vivo studies. AGE exposure impaired the peri‐implant response, whereas AA and AC surfaces consistently promoted a more favorable inflammatory and regenerative environment and improved implant integration under diabetic conditions. Together, these findings support the concept that surface chemistry regulates the biological consequences of AGE‐rich interfaces in vivo. The molecular composition of the adsorbed AGE layer is expected to evolve dynamically within the physiological environment, and direct quantitative analysis of AGEs recovered from explanted biomaterial surfaces would provide additional mechanistic insight into this process. Such studies will further define the relationship between circulating AGE burden, interfacial AGE accumulation and subsequent host responses.

These observations indicate that the pathological influence of diabetes at the implant interface is modifiable, rather than inevitable. Rather than attempting to reduce systemic AGE levels, which remains challenging in clinical practice, our strategy locally rewrites the implant's biological identity. In doing so, we reframe surface engineering from passive biocompatibility improvement to active disease compensation.

The AGE‐pre‐adsorbed implant groups were included to distinguish the contribution of a locally AGE‐rich biomaterial interface from that of the systemic diabetic environment. In healthy animals, AGE pre‐adsorption generated a controlled diabetes‐like interfacial condition in the absence of systemic disease, allowing the effect of local AGE accumulation to be examined independently. In diabetic animals, where systemic metabolic and inflammatory abnormalities are already present, AGE pre‐adsorption provided a more stringent interfacial challenge by superimposing an established local AGE burden on the diabetic host environment. This design therefore allowed us to assess whether additional interfacial AGE loading could further modulate the peri‐implant response and whether the beneficial effects of the engineered surfaces were maintained under an intensified AGE‐rich condition. The inclusion of this group thus provides mechanistic separation between systemic diabetes and local AGE‐mediated effects at the biomaterial interface.

Surfaces that limited AGE binding showed reduced pro‐inflammatory cytokine expression, lower oxidative stress, enhanced reparative mediator expression and promoted M2‐like phenotypes both in vitro and in vivo [52]. The observed modulation of macrophage polarization in this study is consistent with emerging evidence highlighting the importance of immunomodulatory biomaterials in regenerative medicine. Recent studies have demonstrated that promoting the M2 macrophage phenotype can enhance tissue repair under diabetic and inflammatory conditions, including diabetic fracture healing via M2‐derived exosomes [53] and nerve regeneration using electrically conductive scaffolds [54]. The plasma‐polymer coating strategy in the present study modulates the early biomaterial interface by influencing protein adsorption and macrophage responses under AGE‐rich conditions without requiring biological cargo delivery or external stimulation. This improved immune environment was closely associated with reduced fibrosis and restored angiogenesis, two key prerequisites for successful integration.

Moreover, our ability to control the adsorption of AGEs at the biomaterial surface reveals a unifying mechanism explaining how metabolic disease translates into vascular and osteogenic impairment. This translates into clinically important observations, including enhanced neovascularization, reduced capsule thickness, stronger bone–implant contact, and increased osteogenic marker expression, collectively indicating faster recovery of regenerative capacity.

Current strategies to improve implant outcomes for high‐risk populations often rely on systemic management of comorbidities or the addition of antibacterial or osteoinductive cues [55]. Our findings introduce an orthogonal concept that focuses on preventing the formation of a pathological molecular interface. By targeting AGE adsorption, we intervene at the earliest event following implantation, before chronic inflammatory programs are established.

The extended 28‐day stability study provides strong evidence that the biological effects observed at day 30 are not solely due to a transient early modification of the titanium surface. The plasma polymer coatings remained detectable and chemically distinct throughout the experimental period, with only modest changes in thickness, wettability and surface oxidation. These findings support sustained presentation of the engineered interfacial chemistries during the period in which differences in inflammation, vascularization and osseointegration developed in vivo. Although we cannot fully separate the relative contribution of early interfacial conditioning from continued surface activity in vivo, the demonstrated persistence of the coatings strongly supports an ongoing role for the engineered surface chemistry throughout the implantation period.

Several directions for future work warrant consideration. First, although plasma polymer coatings reduced AGE accumulation and improved inflammatory and osseointegration outcomes, the work used titanium‐based substrates and a limited range of implant geometries. While plasma polymerization is substrate‐independent and can be readily applied to metals, polymers, ceramics, and complex 3D architectures, future studies should validate the approach across a broader range of clinically relevant material systems and implant designs, including porous and lattice structures.

Secondly, reduced AGE accumulation on AA and AC surfaces was consistently associated with more favorable immune responses and improved tissue integration. These findings identify interfacial AGE accumulation as an important feature of the biological response to the engineered surfaces. At the same time, surface chemistry regulates a broader range of interfacial properties, including wettability, charge and the composition and organization of the adsorbed protein layer. These physicochemical and biological factors are therefore likely to act in concert rather than independently. Further mechanistic studies examining AGE–RAGE signaling, NF‐*κ*B activation, complement pathways and broader protein‐corona composition will help define how these interconnected interfacial processes contribute to the observed regenerative response.

Thirdly, THP‐1‐derived macrophages provide a well‐established and reproducible model for systematically comparing surface‐dependent macrophage responses under controlled experimental conditions. The consistent phenotypic differences observed between surfaces therefore provide useful mechanistic insight into how surface chemistry influences macrophage behavior. Future studies using primary human macrophages, including cells from donors with and without diabetes, would extend these findings by incorporating donor‐specific variability and the broader metabolic and inflammatory context relevant to patients. Similarly, although the M1/M2 framework and marker panel used here provide a practical means of assessing shifts in macrophage phenotype, macrophage activation in vivo exists across a broader and dynamic phenotypic spectrum. The observed changes are consistent with a shift toward a more reparative macrophage response, and future studies incorporating expanded phenotypic profiling and complementary functional assays will allow this response to be characterized in greater depth.

The clinical serum cohort used in this study was relatively small and was not sex‐balanced, and larger, sex‐balanced cohorts will be important for establishing the broader clinical generalizability of these findings and for examining potential sex‐dependent differences. However, the patient‐derived samples were used primarily to demonstrate that the surface‐dependent AGE adsorption observed in controlled experimental models also occurs in authentic human diabetic serum. Importantly, the same surface‐dependent pattern was consistently reproduced using in vitro glycated serum and two independent AGE‐modified albumin systems, HSA‐AGEs and BSA‐AGEs. This convergence across multiple AGE sources and experimental models provides strong support for surface chemistry as a key determinant of AGE accumulation at biomaterial interfaces.

Additionally, the approximately 30‐day implantation period was selected to capture the early inflammatory response and initial stages of bone integration, which are critical determinants of subsequent implant performance. During this period, the surface modifications clearly improved the local immune environment and early osseointegration under diabetic conditions. Longer‐term implantation studies will be an important next step to determine whether these beneficial responses persist during continued exposure to the diabetic environment and to examine longer‐term bone remodeling, coating stability, fibrotic responses and mechanical fixation under clinically relevant loading conditions. The present findings therefore provide a strong preclinical foundation for the continued development and longer‐term evaluation of these coatings toward clinical translation.

## 4 Conclusion

In summary, this study identifies surface adsorption of advanced glycation end products as a previously unrecognized, disease‐specific mechanism driving impaired implant integration in diabetes. Reduced AGE accumulation on the engineered surfaces was consistently accompanied by improved immune balance, vascularization and tissue integration, supporting interfacial AGE regulation as an important component of the beneficial biological response. These findings establish surface chemistry as a critical determinant of implant performance in high‐risk patients and identify AGE adsorption as a disease‐specific, engineerable target for reprogramming the implant biointerface under diabetic conditions. Looking forward, translating AGE‐resistant surface designs to clinically relevant implant platforms and extending this strategy to other chronic inflammatory and metabolic disease settings may enable the development of next‐generation biomaterials tailored to patient‐specific pathological environments, ultimately improving long‐term clinical outcomes.

## 5 Materials and Methods

### 5.1 Titanium Biomaterials

Grade II commercially pure titanium discs (10 mm diameter, 3 mm thickness) were polished to a surface roughness of Ra 0.5 µm (Hamagawa Industrial (M) SDN BHD, Kedah, Malaysia). Before plasma coating, the discs were sonicated for 8 min in absolute acetone, methanol, ethanol, and distilled water. The discs were then dried with nitrogen and stored in a sterile petri dish. Acrylic acid (C_2_H_3_COOH; AC), allylamine (C_3_H_5_NH_2_; AA), and 1,7‐octadiene (C_7_H_11_CH_3_; OD) (Sigma‐Aldrich, Melbourne, Australia) were used to plasma‐polymerize the titanium surfaces. Non‐coated titanium discs served as negative controls.

### 5.2 Plasma Polymer Coating Deposition

Surfaces were coated in a custom‐built reactor using a previously described 13.56 MHz plasma generator [32]. Plasma air cleaning was performed at 50 W (100%) for 30 min before polymerization. The base pressure reached 2.3×10^−2^ mbar. Ti samples were then placed in the plasma chamber reactor. Air cleaning was then conducted for 5 min at 1×10^−1^ mbar with 50 W (100%). Plasma deposition of AA, AC, and OD was performed at 1.3×10^−1^ mbar for 2 min at 40 W (80%), 10 W (20%), and 20 W (40%), respectively. AC, AA, and OD were selected to span hydrophobic, amine‐rich, and oxygen‐rich plasma chemistries that are widely used to tune interfacial adsorption and cell responses on titanium.

### 5.3 Sessile Drop Water Contact Angle

The water contact angle was determined using an RD‐SDM02 contact angle goniometer (RD Support, Adelaide, Australia). A 50 µL Hamilton microliter syringe (Hamilton Company, NV, USA) was used to place a 5 µL droplet of Milli‐Q water onto the pp‐coated Ti surfaces. The contact angle was measured between the tangent and the surface using the Sessile Drop method. Images were observed and captured using SCA20 Data Physics software (version 6.1). ImageJ analysis software (version 1.54) was used to determine the drop profile, tangent, and angle.

### 5.4 Ellipsometry

After 24 h of plasma polymerization coating, the stability of the pp‐coated surfaces was assessed by measuring the thickness using a SENresearch 4.0 spectroscopic ellipsometer (Sentech Instruments, Berlin, Germany) and WVASE32 software version 2.84 (J.A. Woollam Company, NE, USA). The images and data were processed using SpectraRay/4, a comprehensive ellipsometry software package (SENresearch 4.0).

### 5.5 X‐Ray Photoelectron Spectroscopy (XPS)

A Kratos AXIS Ultra DLD spectrometer operating at 15 keV and 15 mA was used to investigate the surface chemical composition and chemical states of plasma‐coated AC, AA, and OD surfaces. The instrument used a monochromatic Al Kα X‐ray source operating under ultra‐high vacuum conditions. Plasma‐coated silicon wafers were mounted on a sample holder using double‐sided conductive carbon tape to ensure electrical grounding. The survey spectra were recorded in the 0–1100 eV range, with a resolution of 0.5 eV and a pass energy of 160 eV. Additionally, high‐resolution spectra were obtained at a pass energy of 20 eV. Casa XPS software was employed to analyze the data. The binding energy scale was calibrated by adjusting the C 1s peak of adventitious carbon to 284.5 eV.

### 5.6 T2D Patient Samples Used in Ex Vivo Experiments

Serum from normal and T2D patients, along with relevant clinical data, was obtained at Qilu Hospital of Shandong University with informed, written consent. Table S1 presents patient data. The Shandong University Ethics Committee approved this study (Approval Number: KYLL‐2021(KS)‐622). Serum was incubated with the surface ex vivo as previously described. In brief, serum from normal controls and T2D patients was incubated with OD‐, AA‐, and AC‐coated and non‐coated CTL surfaces in a 48‐well plate for at least 2 h at 37°C with agitation at 450 rpm. Subsequently, the surfaces were washed twice with PBS to remove loosely bound proteins. Normal serum served as the control to establish baseline responses for comparison with the experimental conditions.

### 5.7 In Vitro Generation and Quantification of AGEs

To generate AGEs, 10 mL of pooled human serum (HS; #092930149 MP Biomedicals, CA, USA) was incubated with 900 mg of D‐glucose (Merck, Melbourne, Australia) at 37°C, 5% CO_2_ and 95% humidity for 4 to 6 weeks. This prolonged incubation allows glucose to non‐enzymatically glycate serum proteins, mimicking the accumulation of AGEs observed in vivo under hyperglycemic conditions. The serum was dialyzed using Slide‐A‐Lyzer G3 dialysis cassettes (10K MWCO) (ThermoFisher Scientific, MA, USA). A 3 mL sample was loaded into a lyzer device and stored in PBS at 4°C. The buffer solution was replenished every 24 h. AGEs were then collected after 3 d of dialysis. AGEs were stored in a freezer at ‐20°C (Figure S2a). The concentration of AGEs in the serum samples and the number of AGEs adsorbed on the surfaces were measured using the AGE (Advanced Glycation End) Assay Kit (ab238539, Abcam, Cambridge, UK). A dilution series of AGE‐bovine serum albumin (AGE‐BSA) standards, ranging from 0 to 100 µg mL^−1^, was prepared according to the manufacturer's instructions.

### 5.8 Surface Adsorption and Quantification of Serum Proteins

HS was used as a control and incubated with titanium (Ti) samples in a 48‐well plate for 2 h at 37°C with agitation at 450 rpm. Additionally, six‐week in vitro‐generated human serum AGEs (HS‐AGEs) were incubated with Ti samples for 2 h at 37°C under identical conditions. The proteins adhering to the surfaces were gently washed twice with PBS to remove loosely bound proteins. The total protein concentration was measured using a Micro BCA Protein Assay kit (Thermo Scientific, Melbourne, Australia) according to the manufacturer's instructions. Albumin linear standards (0.1–100 ng µL^−1^) were prepared in PBS. The working solution (WS) was prepared at a 25:24:1 ratio of solution A: solution B: solution C. Standards (40 µL) were mixed with 150 µL of WS and 10 µL of SDS–Tris HCl buffer. Samples (10 µL) were mixed with 150 µL of WS and 40 µL of PBS buffer. Incubate standards or samples with WS at 37°C for 2 h. A microplate reader (Synergy HTX multi‐mode reader; Millennium Science Biotek; cat. S1LFTA‐SN; serial no. 22070104) was used to measure absorbance at 570 nm.

### 5.9 Synchrotron ATR‐FTIR Operation and Data Analysis

The spatial distribution of chemical functional groups in human serum‐AGEs (HS‐AGEs) immobilized on different polymers, including AC, AA and OD, was studied using a high‐resolution macro attenuated Total Reflectance (ATR)—Fourier transform infrared (FTIR) mapping instrument. This work was carried out at the Australian Synchrotron (Melbourne, Victoria, Australia) using the infrared microspectroscopy (IRM) beamline, which combines a Bruker Hyperion 3000 Fourier transform infrared (FTIR) microscope with a VERTEX V80v FTIR spectrometer (Bruker Optik GmbH, Ettlingen, Germany) [56]. A 250 µm diameter germanium (Ge) ATR crystal (with a refractive index of nGe  =  4.0) and a 20 × IR objective (with a numerical aperture of NA  =  0.60) were used to reach high brilliance signal‐to‐noise ratios at diffraction‐limited spatial resolutions. Ti samples with pre‐adsorbed AGEs were placed on aluminum discs and inserted into the macro ATR‐FTIR instrument. A spectral range of 3900–950 cm^−1^ and a resolution of 4 cm^−1^ were set in OPUS 8.0 (Bruker). Chemical maps were created by calculating the areas under specific peaks in the spectral data. CytoSpec v. 1.4.02 (Cytospec Inc., Boston, MA, USA) and the UnscramblerX 11.1 software (CAMO Software AS, Oslo, Norway) were used for in‐depth multivariate data analysis.

We mapped the interactions between human serum‐AGEs and three polymers (AA, AC, and OD), focusing on three spectral windows: the methylene region (3000–2800 cm^−^^1^) for lipid changes, the amide region (1700–1600 cm^−^^1^) for protein alterations, and 1200–950 cm^−^^1^ for polysaccharide modifications. Adsorption intensities across these regions were calculated by integrating the signal over each range and are reported in Table S2. The resulting spectral maps show how functional groups distribute within the serum, with shifts indicating structural changes after polymer exposure. To organize the spectral data statistically, we performed hierarchical cluster analysis (HCA) over the protein/polysaccharide range (1810–1000 cm^−^^1^) and lipid range (3004–2810 cm^−^^1^). Spectra were first converted to their second derivative to sharpen band positions, as done in prior studies [57], and five average cluster spectra were extracted. The cluster selected for principal component analysis (PCA) covered the largest area on the map and had the highest peak intensities and lowest noise, particularly in the amide I and C–H stretching bands, the most informative markers in serum. For the comparison between untreated and treated samples, the same HCA approach was applied to the full synchrotron dataset. Each spectrum in the selected cluster underwent second‐derivative transformation followed by extended multiplicative signal correction (EMSC). PCA was then run on the corrected spectra, and PC‐1 and PC‐2 scores were used to map the differences between serum‐AGEs samples before and after exposure to the immobilized polymers.

### 5.10 Cell Culture and Differentiation

The human monocyte‐derived cell line, THP‐1 cells (SKU: 88081201, Sigma‐Aldrich, MO, USA), were cultured in RPMI‐1640, no glucose medium (Sigma‐Aldrich, MO, USA), supplemented with 4.5 mmol L^−1^ (w/v) of glucose, 10% (v/v) FBS, 1% (v/v) penicillin (100 U mL^−1^), and streptomycin (100 µg mL^−1^). The culture medium was replaced every 2–3 d. THP‐1 cells were differentiated into macrophage‐like cells (dTHP‐1) at passage 14 by treatment with phorbol‐12‐myristate‐13‐acetate (PMA, 100 ng mL^−1^, Sigma‐Aldrich, MA, USA) for 48 h at 37 °C, with 5% CO_2_ and 95% humidity. The PMA‐containing media were then removed, and the cells were rested in normal media for 24 h at 37°C, 5% CO_2_ and 95% humidity and then maintained in low‐glucose Dulbecco's modified Eagle's medium (DMEM) (Gibco, Melbourne, Australia), containing 10% (v/v) fetal bovine serum (FBS) and 1% (v/v) penicillin‐streptomycin, before use. After polarization, populations of M1 and M2 on different subsamples were then analyzed spectroscopically, flow cytometrically, and molecularly.

### 5.11 Flow Cytometry for Cell Viability and Apoptosis

Flow cytometry data were acquired on a CytoFLEX S instrument (Beckman Coulter, CA, USA) and analyzed using CytExpert software. The proportions of viable, apoptotic, and dead cells were assessed by staining cells with FITC‐conjugated Annexin V and Propidium Iodide (PI) using the BD Pharmingen Annexin V‐FITC Apoptosis Detection Kit (#556547, BD Biosciences, NJ, USA). Cells that were negative for both Annexin V and PI were considered viable. Annexin V‐positive/PI‐negative cells are early apoptotic, while Annexin V‐positive/PI‐positive cells are dead. Cells were seeded at a density of 1 × 10^6^ cells per well on plasma‐polymerized model surfaces (AC, AA, and OD) and on either HS‐ or HS‐AGE‐coated surfaces in 24‐well plates and cultured for up to 21 d. Cells were washed with PBS, detached with trypsin, and pelleted by centrifugation at 1,000 rpm for 3 min. Cells were resuspended in 300 µL of 1× Annexin V binding buffer. 5 µL of Annexin V‐FITC (Ex/Em = 488/530 nm) and 5 µL of PI (Ex/Em = 561/610 nm) were added, then incubated for 15 min at room temperature in the dark.

In vitro label‐free characterization of classically (M1) and alternatively (M2) polarized monocyte‐derived macrophages (MDM) was performed by staining with monoclonal mouse anti‐human CD80^+^ M1 (ab27554, Abcam, Cambridge, UK) and CD206^+^ M2 FITC Anti‐mannose receptor (ab270647, Abcam, Cambridge, UK) antibodies. MDMs were washed with PBS, detached from the surfaces using 0.25% trypsin, and pelleted by centrifugation at 1,000 rpm for 3 min. Cells were resuspended in 300 µL of PBS. Five µL of anti‐human CD80^+^ M1 (Ex/Em: 650/660 nm) or 5 µL of FITC CD206^+^ M2 (Ex/Em: 495/519 nm) were added, followed by a 15 min incubation at room temperature in the dark. Non‐specific antigens were blocked with 5% BSA in PBS. Cells were analyzed using a BD FACSCanto II Flow Cytometer (BD Biosciences). Non‐specific mouse IgGs were used as an isotype control. Counted events ranged from 10,000 to 20,000. Debris and cell aggregates were excluded from the analysis using forward scatter (FSC) and side scatter (SSC) parameters, and single cells were gated using FSC‐area scaling. Positive staining and autofluorescence were determined using unstained cells as a negative control.

### 5.12 Scanning Electron Microscopy

The topography of plasma‐polymer‐coated Ti surfaces and uncoated Ti surfaces was assessed using scanning electron microscopy (SEM). Cells were fixed and dehydrated as follows. First, cells were fixed in 4% glutaraldehyde in 0.1 m sodium cacodylate buffer (pH 7.4) for 40 min (10 × 10 mL each; Electron Microscopy Sciences, PA, USA). The samples were washed twice with PBS for 5 min per wash, then dehydrated through a graded ethanol series from 30% to 100% (v/v), with two changes at each concentration for 10 min per change. The samples were immersed in a 1:1 (v/v) mixture of ethanol and hexamethyldisilazane (HMDS; Merck, Germany) for 15 min, followed by pure HMDS for 20 min. The HMDS was then discarded, and the samples were air‐dried in a fume hood for at least 2 h. After mounting on aluminum stubs with double‐sided carbon tape, the samples were sputter‐coated with a 5 nm platinum layer using a K575X Emitech sputter coater (PolyK Technologies, PA, USA). Surface morphology was examined using an FEI Inspect F50 scanning electron microscope (FEI Company, Oregon, USA) at an accelerating voltage of 10 kV and a working distance of 5 mm. Images were acquired at 1024 × 1024 pixels.

### 5.13 Confocal Microscopy

Surfaces pre‐adsorbed with AGEs were incubated with FITC‐conjugated anti‐AGE antibody (ab23722, ref A64443, Abcam, Cambridge, UK) at room temperature for 30 min. Following staining with the FITC‐conjugated anti‐AGE antibody, samples were washed twice with PBS for 10 min per wash to remove unbound and loosely retained antibody and minimize background fluorescence prior to imaging. The surfaces were stored in PBS for confocal microscopy to visualize adsorbed AGEs. To improve penetration, the cells were incubated in 300 µL of 0.1% (v/v) Triton X‐100 for 10 min. The cells were then washed once in PBS. The cytoskeleton was stained with a 1:400 dilution of Alexa Fluor 488‐conjugated phalloidin in PBS (Sigma, Melbourne, Australia) (excitation/emission: 495/518 nm) for 30 min at room temperature in the dark. Subsequently, using PBS to wash and the nuclei were washed with PBS and stained using a 1:1000 dilution of 4',6‐diamidino‐2‐phenylindole (DAPI, excitation/emission: 365/465 nm, 0.1 µg mL^−1^, Invitrogen, Life Technologies, CA, USA) for 5 min. Lastly, DAPI was discarded, and the cells were stored in PBS for Confocal Microscopy.

A Zeiss LSM 880 system (Zeiss, Oberkochen, Germany), equipped with Fast Airyscan and an argon‐ion laser, and running ZEN Black 3.3 software, was used to visualize the cells. The sample was placed on a glass‐bottom tissue culture dish (Dish ø 35 mm, Glass ø 23 mm, Glass thickness 0.17 mm, World Precision Instruments, Inc., Australia) for microscopy. The images were captured using a Plan‐Apochromat 20x/0.8 M27 objective in both the Phalloidin (Alexa* Fluor 488) and DAPI channels. The pixel size was 0.15 × 0.15 µm, and the z‐axis interval was 0.5 µm, enabling optical sectioning. Cells attached to engineered surfaces were imaged at three randomly selected areas at 40X magnification. Cell counts were performed using ImageJ (version 1.54).

### 5.14 Oxidative Stress Detection

To assess intracellular oxidative stress in cells cultured on the model Ti surfaces, we seeded 5 × 10^3^ dTHP‐1 macrophages onto each surface in a 48‐well plate and incubated them at 37°C, 5% CO_2_, and 95% humidity for 24 h. After incubation, remove the media and incubate the cells in 200 µL of 10 nmol L^−1^ 2′,7′‐dichlorofluorescein (DCF, Sigma, MO, USA) in PBS for 30 min at 37°C in 5% CO_2_. Cells were then washed with PBS and stained with DAPI (1 nmol L^−1^) for 15 min. Stained cells were stored in PBS and visualized under a Zeiss LSM 880 system (Zeiss, Jena, Germany). ROS‐activated DCF fluorescence was visualized at 490 nm excitation and 520 nm emission. DCF channels were set to 501 – 627 nm in the confocal software. The fluorescence intensity was quantified using ImageJ software (version 1.54; NIH, MD, USA). First, we opened “Set Measurements” from the Analyze menu and ensured “integrated density” and “mean grey value” were selected. Next, the cells of interest were selected using the drawing/selection tools (i.e., rectangle, circle, or polygon). Then, the “measure” option was selected, and the background and cellular fluorescence values were recorded. Finally, all the data were copied and pasted into an Excel spreadsheet and analyzed using the formula below to calculate the corrected total cell fluorescence (CTCF). CTCF = integrated density—(area of selected cell x mean fluorescence of background readings).

### 5.15 RNA Extraction

RNA was extracted from dTHP‐1 macrophages using the QIAwave RNA Mini Kit (250) (QIAGEN, Hilden, Germany) according to the manufacturer's instructions. The extracted RNA was then eluted in 50 µL of elution buffer and stored at –80°C for future analyses. To ensure accurate PCR results, a negative control (reagent blank) was included in all subsequent PCR analyses to eliminate contamination and false positives.

### 5.16 Quantitative Polymerase Chain Reaction (qRT‐PCR)

RNA quantity and quality were assessed using a NanoDrop One/OneC Microvolume UV–Vis Spectrophotometer (Thermo Fisher Scientific, MA, USA). One‐step qRT‐PCR enables cDNA synthesis and PCR in a single tube with gene‐specific primers. The GoTaq 1‐Step RT‐qPCR System (5 mL; Promega Corporation, USA; catalog number A6020) was used. Amplification was conducted using the Rotor‐Gene Q real‐time PCR cycler (Qiagen, Venlo, Netherlands) under the following conditions: 50°C for 3 min, followed by 95°C for 5 min, and then 40 cycles of denaturation at 95°C for 15 s and annealing and extension at 60°C for 30 s. Fluorescence was measured at 60°C. A melting curve was generated between 72°C and 95°C in 1°C increments to determine amplification specificity. The threshold cycle number (Ct) values were calculated for each reaction. The relative expression of each target mRNA was estimated using the Livak method (2‐ΔΔCt) [58] and normalized to the housekeeping genes glyceraldehyde‐3‐phosphate dehydrogenase (GAPDH) or Beta‐actin (ACTB), yielding fold changes for each target gene. The supplementary data provide the primer sequences (Table S3).

### 5.17 Surface‐immobilized AGEs and the Absence of Soluble AGEs

Ti control surfaces (*n* = 3) were pre‐adsorbed with AGEs for 2 h and subsequently washed twice with PBS to remove unbound proteins. AGE‐pre‐adsorbed Ti surfaces and corresponding Ti control surfaces without AGE adsorption were then incubated in DMEM at 37°C, 5% CO_2_ and 95% humidity for 24 h. The resulting conditioned media were collected and used to assess the biological activity of any AGE‐modified species released from the surfaces. Macrophage‐like dTHP‐1 cells were seeded at 5 × 10^3^ cells per well in 48‐well plates and exposed for 24 h to conditioned medium collected from either AGE‐pre‐adsorbed or control Ti surfaces under otherwise identical culture conditions.

### 5.18 In Vivo Study

#### 5.18.1 Chemicals

The hematoxylin and Eosin (H & E) Staining Kit, Masson's trichrome staining kit, DAB Kit, Immunofluorescence Staining Kit, and QuickBlock Secondary Antibody Dilution Buffer for Immunohistochemistry and Immunolabeling Staining Primary Antibody Dilution Buffer were purchased from Beyotime (China). Streptozotocin (STZ) was obtained from MCE (China). Antibodies to *VEGFA*, CD31, Osteocalcin, *Runx2*, *TNF‐α*, *IL‐10*, CD206, and *iNOS* were purchased from Sigma‐Aldrich.

#### 5.18.2 Animal Model

The Ethics Committee of Shandong University approved all experimental protocols (Approval Number: DWLL‐202500205), and the study followed the Guidelines for the Management and Use of Laboratory Animals. Male Sprague‐Dawley rats (6‐8 weeks old) were stratified into healthy and diabetic cohorts, with each cohort further randomized into four subgroups: Control, OD, AA, and AC. Sample size varied by experimental endpoint. Micro‐CT analysis, H&E histology, VEGFA immunohistochemistry, and CD31, CD206, and iNOS immunofluorescence included six animals per subgroup. Masson's trichrome analysis of fibrous capsule thickness and immunohistochemistry for OCN, Runx2, TNF‐α, and IL‐10 included four animals per subgroup.

Following a one‐week acclimatization period, diabetic rats were fed a high‐fat/high‐sucrose diet for 6 weeks and fasted for 12 h. STZ (1% solution in citrate buffer, 30 mg kg^−1^ body weight) was administered via intraperitoneal injection to induce diabetes, whereas normoglycemic controls received an equivalent volume of sterile saline. Fasting blood glucose levels were quantified 24 h post‐injection; values exceeding 16.7 mmol L^−1^ confirmed successful establishment of the diabetic model. A standardized surgical site was selected on the lateral plane of the femoral condyle. Under aseptic conditions and deep anesthesia, a 1.5‐mm diameter drill was used to create a bicortical defect at the midpoint of the femoral condyle. An implant rod (10 mm length × 1.3 mm diameter) was vertically inserted into the distal femur. Layered incision closure was performed, and all animals received postoperative intramuscular antibiotics for 72 h. Euthanasia was conducted 30 d postoperatively for tissue harvesting. Rats were anesthetized via intraperitoneal injection of pentobarbital sodium (50 mg kg^−1^). After removing dorsal hair and disinfecting the skin, a 1.0‐cm longitudinal incision was made along the midline. Prefabricated implants were then inserted into the subcutaneous pocket, and the wound was carefully closed.

#### 5.18.3 Blood Glucose Measurement

Following model establishment, blood samples were collected from the tail vein every 3 d, and blood glucose was measured using a glucometer. We defined successful diabetes induction as a random blood glucose level ≥16.7 mmol L^−1^. Blood glucose levels were subsequently monitored in all material treatment groups.

#### 5.18.4 Measurement of Serum Advanced Glycation End Products

At the end of the experiment, rats were fasted for 12 h with free access to water, weighed, and anesthetized with isoflurane. Blood was collected from the retro‐orbital venous plexus into tubes containing a clot activator and allowed to clot at room temperature for 30 min. Samples were then centrifuged at 3000 rpm for 15 min at 4°C. Serum was aliquoted and stored at −80°C until analysis. Serum concentrations of advanced glycation end products (AGEs) were measured using a rat AGEs ELISA kit (FineTest, ER0268) according to the manufacturer's instructions. Absorbance was measured at 450 nm, and AGEs concentrations were determined from the standard curve.

#### 5.18.5 Micro‐CT Analysis

After dissecting the femoral muscle tissue, the implant‐containing femurs were fixed in 10% neutral buffered formalin for 12 h at 4°C. To perform radiographic evaluation, specimens were scanned using a SkyScan 1276 high‐resolution micro‐computed tomography (µCT) system (Bruker, MA, USA) under the following acquisition parameters: scanning layer thickness 15 µm, X‐ray tube voltage of 70 kV, current of 500 µA and scanning range 2.5 cm × 2.5 cm. A standardized region of interest (ROI) extending 200 µm circumferentially around the implant was defined using CTAn software (Bruker microCT, version 1.18.8.0). Quantitative morphometric analysis was performed, including the following parameters: bone mineral density (BMD, g cm^−^^3^) (*n* = 6), bone volume fraction (BV/TV, %) (*n* = 6), and trabecular number (Tb.N, mm^−^^1^) (*n* = 6). 3D reconstructions were generated using Mimics software (Version 21.0, Materialise, Belgium) and CTVox software (Bruker microCT, version 3.3.0 r1403).

#### 5.18.6 Histological, Immunohistochemical, and Immunofluorescence Staining

After being harvested, the skin was rinsed in sterile 0.9% sodium chloride, immersion‐fixed in 4% paraformaldehyde (pH 7.4), and subjected to gradient ethanol dehydration. Paraffin‐embedded specimens were sectioned at 4 µm thickness. Histological evaluation was performed using hematoxylin‐eosin (H&E) staining to quantify inflammatory cell infiltration (*n* = 6) and Masson's trichrome staining to measure the thickness of the fibrous layers (*n* = 4) from normal and diabetic rats. Femoral specimens were fixed in 10% neutral buffered formalin for 48 h, decalcified in 14% EDTA (pH 7.2) for 21 d, and then processed through paraffin embedding. The sections were analyzed following H&E staining to assess the tissue microarchitecture (*n* = 6). Immunohistochemistry was performed using primary antibodies against *VEGFA* (0.2 mg mL^−1^) (1:200) in cutaneous tissue (*n* = 6), and *OCN* (2.73 mg mL^−1^) (*n* = 4), *Runx2* (1 mg mL^−1^) (*n* = 4), *TNF‐α* (1 mg mL^−1^), and *IL‐10* (1 mg mL^−1^) (1:100) in osseous tissue (*n* = 4). After dewaxing and gradient hydration of the tissue sections, we added primary antibodies against VEGFA, OCN, Runx2, TNF‐α, or IL‐10 and incubated the sections overnight at 4°C. Then, we incubated the sections with the corresponding secondary antibody for 30 min. The protein of interest was detected using a 3,3’‐diaminobenzidine (DAB) kit (Biyuntian, China). Quantification was performed by calculating the density of positive cells in six randomly selected fields at 200× magnification. Immunofluorescence staining was performed using anti‐CD31 (1 mg mL^−1^) (1:150) for dermal vasculature and anti‐CD206 (1 mg mL^−1^)/*iNOS* (2 mg mL^−1^) (1:200) to assess macrophage polarization (*n* = 6). The paraffin‐embedded tissue sections were rehydrated, then heated at 60°C for 1 h. Subsequently, antigen retrieval and blocking were performed. Primary antibodies to CD31, CD206, and *iNOS* were then added, and the sections were incubated overnight at 4°C. The sections were washed and then incubated with appropriate fluorochrome‐conjugated antibodies at room temperature for 1 h. After incubation with DAPI (1 nmol L^−1^), the slices were sealed with an anti‐fade sealing agent and evaluated by confocal fluorescence microscopy. Fluorescence intensity for each protein was assessed using ImageJ software (version 1.54).

### 5.19 Statistical Analysis

All experimental details, including the number of experiments (n), mean, and standard deviation (SD), are provided in the Figure legends. An ordinary one‐way ANOVA followed by Tukey's multiple comparison post hoc test was used to determine significant differences among more than two groups, and the Mann‐Whitney *U*‐test was used as a non‐parametric independent test for comparing the means of two groups. All tests were performed using GraphPad Prism (version 9.5.1) software [59]. A significance level of *p* < 0.05 was considered statistically significant.

## Author Contributions

Conceptualization: R.B. and K.V. Methodology: T.N.L., W.C., and T.H. Visualization: T.N.L., R.B., W.C., T.H., T.T.N., J.V., M.N.A., X.D.D., V.K.T., G.B., Y.Z., W.L., and K.V. Investigation: T.N.L., W.C., T.H., T.T.N., J.V., M.N.A., X.D.D., G.B., R.B., Y.Z., W.L., and K.V. Funding acquisition: R.B., Y.Z., W.L., and K.V. Project administration: T.N.L., W.C., and T.H. Supervision: R.B., Y.Z,, W.L., and K.V. Writing – original draft: T.N.L., W.C., T.H., T.T.N., J.V., G.B., R.B., and K.V. Writing – review & editing: T.N.L., R.B., W.C., T.H., T.T.N., J.V., G.B., Y.Z., W.L., and K.V.

## Funding

K.V. thanks NHMRC for the grant GNT1194466. K.V. and R.B. thanks ARC for grant DP250101028. R.B. thanks Diabetes Australia for the grant DAP‐Y2600044. V.K.T. thanks ANSTO for funding the beamline at the Australian Synchrotron. Y.Z. and W.L. thank the National Natural Science Foundation of China (Grant No. 82072478 to Yunpeng Zhao; Grant No. 82073437 to Weiwei Li) and the Shandong Provincial Natural Science Foundation (Grant No. ZR2020YQ54 to Yunpeng Zhao) for funding.

## Ethics Statement

We confirm that we have complied with all relevant ethical regulations, as detailed in the Methods.

## Conflicts of Interest

The authors declare no conflicts of interest.

## Supporting information




**Supporting File**: advs78507‐sup‐0001‐SuppMat.docx.

## Data Availability

The experimental data supporting this study's findings are available in the Supporting Information.
