# Supplementary material for: Rational Surface Engineering Reduces Advanced Glycation End‐Product Adsorption and Improves Implant Integration
Source: Adv Sci (Weinh). 2026 Oct 27:e78507. Online ahead of print. doi: 10.1002/advs.78507 (PMC13712394; doi:10.1002/advs.78507)
Supplement: Supplementary file 1 [file ADVS-9999-e78507-s001.docx]

**Supplementary Material**

**Rational surface engineering reduces advanced glycation end-product adsorption and improves implant integration**

Thien Ngoc Le^1,†^, Wenhao Chen^2, †^, Tengxiao Huang^2, †^, Tien Thanh Nguyen^1^, Markos Negash Alemie^1^, Xuan Duy Do^1^, Giles Best^3^, Jordan Li^4^, Jitraporn Vongsvivut^5^, Vi-Khanh Truong^1^, Weiwei Li^6*^, Yunpeng Zhao^2*^, Richard Bright^1*^, Krasimir Vasilev^1*^

^1^Biomedical Nanoengineering Lab, College of Medicine and Public Health, Flinders University, South Australia 5042, Australia

^2^Department of Orthopaedics, Qilu Hospital, Cheeloo College of Medicine, Shandong University, Jinan, Shandong, 250012, PR China

^3^Flow Cytometry facility, Health and Medical Research Building, College of Medicine and Public Health, Flinders University, South Australia 5042, Australia

^4^Department of Renal Medicine, Flinders Medical Centre, South Australia 5042, Australia

^5^Infrared Microspectroscopy (IRM) Beamline, Australian Synchrotron, Victoria 3168, Australia

^6^Department of Pathology, Qilu Hospital, Cheeloo College of Medicine, Shandong University, Jinan, China

† These authors contributed equally.

*Corresponding author.

[richard.bright@flinders.edu.au](mailto:richard.bright@flinders.edu.au); [krasimir.vasilev@flinders.edu.au](mailto:krasimir.vasilev@flinders.edu.au); [lwwzyp@email.sdu.edu.cn](mailto:lwwzyp@email.sdu.edu.cn); [liweiweizhao@163.com](mailto:liweiweizhao@163.com)

Plasma-coated samples (OD, AA and AC) were incubated in PBS at 37 °C for up to 28 days to assess surface stability. Coating thickness and WCA were measured at days 0, 1, 7, 14 and 28, with three replicates per condition. Surface chemical composition was assessed by XPS at days 0 and 28, including elemental composition and high-resolution C 1s spectra.

**Figure S1**. Surface coating thickness measured at D0, D1, D7, D14, and D28. Data are presented as mean ± SD and *n* = 3.

**Figure S2.** Water contact angles of surface coatings measured at D0, D1, D7, D14, and D28. Data are presented as mean ± SD and *n* =3

**Figure S3.** High-resolution XPS C1s survey spectra for the three coatings, measured at day 0 and day 28.

**Figure S4.** Cellular responses induced by surface-immobilized AGEs. Advanced Glycation End Products (AGEs) immobilized on the biomaterial surface reprogram immune responses by binding to the receptor for AGEs (RAGE) on the cell membrane, initiating downstream signaling cascades that drive apoptosis and pro-inflammatory activation. These surface-mediated processes impair immune regulation and contribute to diabetes-related complications.

**Figure S5**. Generation and quantification of AGEs. (**a**) Schematic representation of the protocol for generating AGEs in vitro (HS-AGEs). (b-d) FITC-conjugated anti-AGE antibody-based quantification of AGEs in (**b**) normal human serum and type 2 diabetic (T2D) patients’ serum, n = 8; (**c**) human serum albumin (HSA) and in vitro 6-week AGEs (HSA-AGEs), *n* =3; (**d**) bovine serum albumin (BSA) and commercial AGEs (BSA-AGEs), *n* =3. (**e**) Quantification of total protein adsorption on the surfaces using the micro‑BCA assay showed enhanced interaction with normal serum proteins (*n* = 5). (**f**) Confocal fluorescence microscopy images show reduced AGEs accumulation on AA- and AC-coated surfaces after incubation with either diabetic patients’ serum, HSA, HSA-AGEs, BSA or BSA-AGEs. The scale bar represents 100 µm. (**g**) FITC-conjugated anti-AGE antibody-based quantification of AGEs absorbed on different surfaces after incubation with human serum, T2D patient serum or *in vitro* HSA, HSA-AGEs or BSA and BSA-AGEs. Data are presented as mean ± SD, *n* = 3, **p* < 0.05, ***p* < 0.01, ****p* < 0.001 and *****p* < 0.0001.

**Figure S6.** AGE-resistant effect of responsive surfaces on the proliferation of immune cells. (**a**) Flow cytometry assessed the viability of human monocyte THP-1 cells using Annexin V-FITC and/or Propidium Iodide (PI); Annexin V/PI-negative cells were considered viable. The data show fewer viable cells and more apoptotic cells on surfaces pre-adsorbed with serum from T2D patients and HS-AGEs. (**b**) The proportion of viable THP-1 monocytes cultured on surfaces pre-adsorbed with ex vivo or in vitro serum. (**c**) The proportion of apoptotic THP-1 monocytes cultured on surfaces pre-adsorbed with serum. Apoptosis decreased on AC surfaces pre-adsorbed with T2D patient and HS-AGEs serum to levels below those in normal serum. (**d**) Representative flow cytometry cytograms of dTHP-1, highlighting differences in the proportions of viable and apoptotic cells between the two study groups. (**e**) The proportion of viable dTHP-1 cells cultured on the surfaces with pre-adsorption of *ex vivo* or *in vitro* serum. (**f**) The proportion of apoptotic dTHP-1 cells on the surfaces with pre-adsorption of *ex vivo* or *in vitro* serum. Data are presented as mean ± SD and *n* =3.

**Figure S7.** Impact of *ex vivo* AGEs accumulation on macrophage polarization. (a) Confocal fluorescence microscopy images depicting the polarisation of dTHP-1 cells on surfaces with different chemical modifications after incubation in normal and T2D patient serum. Note the anti-inflammatory M2 phenotype cell morphology observed on AA and AC coatings. Scale bar: 10 µm. (b, c) Flow cytometry analysis detecting (b) CD80+ M1-like macrophages and (c) CD206+ M2-like macrophages cultured on the functional surfaces (*n* = 3).

**Figure S8.** AGE-resistant surface effect promotes anti-inflammatory responses. (a) Fluorescence microscopy images show intracellular ROS levels in macrophages after 24 h of culture on surfaces with different chemical modifications, after incubation with HS and in vitro-generated HS-AGEs. The scale bar represents 50 µm. (b) Quantification of intracellular ROS levels from the images in (a). Note the significant reduction in intracellular ROS levels on AC and AA surfaces. (c, e) Representative flow cytometry analysis detecting (c) CD80+ M1-like macrophages and (e) CD206+ M2-like macrophages. (d, f) Quantification of (d) M1-like and (f) M2-like cell populations cultured on the different surfaces, indicating a significantly higher number of M2 and lower M1 cells recorded on the AC and AA coatings post-incubation in HS and HS-AGEs. (g and h) Gene expression heatmap showing upregulated mRNA levels of (g) pro-inflammatory cytokines IL-1β, TNF-α, IL-6, and iNOS; and (h) anti-inflammatory cytokines IL-1RA, ARG-1, IL-10, and TGF-β, produced by macrophages cultured on the engineered surfaces after incubation in HS and HS-AGEs. Data are presented as mean ± SD, *n* = 3, **p* < 0.05, ***p* < 0.01, ****p* < 0.001 and *****p* < 0.0001.

**Figure S9.** Inflammatory responses induced by soluble AGEs. Pro‑inflammatory cytokines (IL‑1β, TNF‑α, IL‑6) production by macrophages exposed to supernatants collected after incubation with AGE-adsorbed and CTL surfaces. Data are presented as mean ± SD (n = 9).

**Figure S10**. Line graphs showing glucose and serum AGEs levels in healthy and diabetic rats at specific time points over 30 days.

**Figure S11.** Anti-inflammatory responses in peri-implant bone tissue of healthy and diabetic rats with the presence of AGEs (AGE (+)). (a) Representative images of bone tissue from normoglycemic rats and (c) diabetic rats stained with antibodies to *TNF-α* and *IL-10*. Scale bars represent 50 μm. (b) Quantification of *TNF-α* and *IL-10* expression in healthy rats (*n* = 4). (c) Representative images of bone tissue from diabetic rats stained with antibodies to *TNF-α* and *IL-10*. Scale bars represent 50 μm. (d) Quantification of *TNF-α* and *IL-10* expression in diabetic rats (*n* = 4). Data are plotted as mean ± SD. Statistical significance: **p* < 0.05, ***p* < 0.01, ****p* < 0.001 and *****p* < 0.0001.

**Table S1**. Demographic and clinical characteristics of patients with T2D and controls (n = 8 per group). Data are presented as mean ± standard deviation or participant counts for sex. Blood pressure is reported as systolic/diastolic pressure. p-values indicate comparisons between groups. NS, not statistically significant; HDL, high-density lipoprotein; LDL, low-density lipoprotein; AGEs, advanced glycation end products. Dashes indicate not applicable or not assessed.

| **Demographics** | **T2D patients** | **Controls** | ***p-*value** |
| --- | --- | --- | --- |
| Sex, female/male | 2 / 6 | 6 / 2 | - |
| Age (years) | 61.80 ± 11.30 | 59.50 ± 7.90 | *NS* |
| Disease duration (years) | 6.90 ± 8.40 | - | - |
| Fasting plasma glucose, mmoL/L | 5.90 ± 1.20 | 4.80 ± 0.46 | 0.029 |
| Glycated albumin, % | 17.10 ± 3.50 | 13.50 ± 1.92 | 0.023 |
| Body mass index, kg/m^2^ | 26.10 ± 2.20 | 25.40 ± 2.70 | NS |
| Blood pressure, mmHg | 136±13/79±8 | 129±13/81±9 | NS |
| White blood cell count, 10^9^/L | 5.90 ± 1.30 | 5.10 ± 2.30 | NS |
| Total cholesterol, mmoL/L | 4.16 ± 1.44 | 4.42 ± 0.73 | NS |
| HDL cholesterol, mmoL/L | 1.15 ± 0.32 | 1.25 ±0.20 | NS |
| LDL cholesterol, mmoL/L | 2.44 ± 1.08 | 2.71 ± 0.60 | NS |
| Triglycerides, mmoL/L | 1.46 ± 0.66 | 1.32 ± 0.48 | NS |
| Level of AGEs, ng/mL | 44.91 ± 6.36 | 30.34 ± 3.96 | <0.0001 |

NS indicates not significant (*p* > 0.05). AGEs were measured by immunoassay and reported as units (ng/mL), reflecting relative antibody-binding intensity rather than absolute mass concentration.

**Table S2**. Summary of significant wavenumbers ^1^ detected in the loading plots for HS-AGEs absorbed on the surfaces.

| **Wavenumber value**  (cm^-1^) | **Band assignment** |
| --- | --- |
| **Lipid** |  |
| ~2925  ~2951  ~2953 | ѵCH_2_ of lipids  Stretching C-H  Asymmetric CH_2_ stretching mode of the methylene chains in membrane lipids |
| ~2838 | Stretching C-H |
| **Protein** |  |
| **Amide I** |  |
| ~1664  ~1658 | C=O: cytosine, uracil  C=O, stretching C=C uracil, NH_2_ guanine |
| ~1638  ~1601  ~1632 | C=C thymine, adenine, N-H guanine  C=N cytosine, N-H adenine  Ring C-C stretch of phenyl |
| **Amide II** |  |
| ~1514 | n(C=C)-diagnostic for the presence of a carotenoid structure, most likely a cellular pigment |
| ~1526  ~1537  ~1544  ~1559  ~1576 | C=N guanine  Stretching C=N, C=C  C-N stretching & C-N bending vibrations  Ring base  C=N adenine |
| ~1492 | Deformation C-H |
| ~1446  ~1420 | Asymmetric CH_3_ bending of the methyl groups of proteins  Deformation C-H |
| ~1374 | Stretching C-N cytosine, guanine |
| ~1396  ~1312 | Symmetric CH_3_ bending of the methyl groups of proteins  Amide III band components of proteins |
| **Amide III** |  |
| ~1238 | Asymmetric PO_2_־ stretching, collagen |
| ~1204  ~1196  ~1106  ~1094 | Vibrational modes of collagen proteins-amide III, C-O-C, C-O dominated by the ring vibrations of polysaccharides C-O-P, P-O-P  Collagen  Carbohydrates  ѵ (C-O-C) (polysaccharides-cellulose) |
| ~1088 | Phosphate I (stretching PO_2_־ symmetric vibration) in *β*-form DNA |
| ~1053  ~1035 | ѵC-O & ꝽC-O of carbohydrates  Glycogen ѵ(CO), ѵ(CC), ѵ(CCO), (polysaccharides cellulose) |

**Table S3**. List of primer pairs used for real-time quantitative PCR.

| Name | GenBank ID | Primer set | Sequence | Tm (°C) | Product size (bp) |
| --- | --- | --- | --- | --- | --- |
| Housekeeping gene |  |  |  |  |  |
| Glyceraldehyde-3-phosphate dehydrogenase | AF261085.1 | GADPH_F | CACCCACTCCTCCACCTTTG | 84 | 110 |
|  |  | GADPH_R | CCACCACCCTGTTGCTGTAG |  |  |
| Actin beta | NM_001101.5 | ACTB_F | CACCATTGGCAATGAGCGGTTC | 85 | 135 |
|  |  | ACTB_R | AGGTCTTTGCGGATGTCCACGT |  |  |
| Pro-inflammatory cytokine |  |  |  |  |  |
| Interleukin 1 beta | NM_000576.3 | IL-1β_F | TGATGGCTTATTACAGTGGCAA | 87 | 185 |
|  |  | IL-1β_R | GTCCATGGCCACAACAACTG |  |  |
| Tumor necrosis factor alpha | NM_000594.4 | TNFα_F | CCTCTCTCTAATCAGCCCTCTG | 88 | 220 |
|  |  | TNFα_R | GAGGACCTGGGAGTAGATGAG |  |  |
| Interleukin 6 | M54894.1 | IL6_F | AGACAGCCACTCACCTCTTCAG | 83 | 132 |
|  |  | IL6_R | TTCTGCCAGTGCCTCTTTGCTG |  |  |
| Nitric oxide synthase, inducible | L24553.1 | iNOS_F | GCTCTACACCTCCAATGTGACC | 86 | 136 |
|  |  | iNOS_R | CTGCCGAGATTTGAGCCTCATG |  |  |
| Anti-inflammatory cytokine |  |  |  |  |  |
| Arginase 1 (ARG1) | NM_000045.4 | ARG1_F | TCATCTGGGTGGATGCTCACAC | 82 | 130 |
|  |  | ARG1_R | GAGAATCCTGGCACATCGGGAA |  |  |
| Interleukin 1 receptor antagonist | NM_173841.3 | IL-1RA_F | ATGGAGGGAAGATGTGCCTGTC | 84 | 104 |
|  |  | IL-1RA_R | GTCCTGCTTTCTGTTCTCGCTC |  |  |
| Interleukin 10 | CR542028.1 | IL10_F | TCTCCGAGATGCCTTCAGCAGA | 83 | 126 |
|  |  | IL10_R | TCAGACAAGGCTTGGCAACCCA |  |  |
| Transforming growth factor beta 1 | XM_054321897.1 | TGF- β_F | TACCTGAACCCGTGTTGCTCTC | 84 | 122 |
|  |  | TGF- β_R | GTTGCTGAGGTATCGCCAGGAA |  |  |

**Table S4**. Fasting blood glucose levels of healthy and diabetic rats.

**Table S5**. Serum AGE levels of healthy and diabetic rats.
